# Hypofractionated radiotherapy combined with lenalidomide improves systemic antitumor activity in mouse solid tumor models

**DOI:** 10.7150/thno.88864

**Published:** 2024-04-08

**Authors:** Kateryna Onyshchenko, Ren Luo, Xi Rao, Xuanwei Zhang, Simone Gaedicke, Anca-Ligia Grosu, Elke Firat, Gabriele Niedermann

**Affiliations:** 1 Department of Radiation Oncology, Faculty of Medicine, University of Freiburg, Freiburg, Germany.; 2 Faculty of Biology, University of Freiburg, Freiburg, Germany.; 3 Laboratory of Biosynthesis of Nucleic Acids, Institute of Molecular Biology and Genetics of NASU, Kyiv, Ukraine.; 4 German Cancer Consortium (DKTK), Partner Site Freiburg, Freiburg, Germany.; 5 German Cancer Research Center (DKFZ), Heidelberg, Germany.; 6 Division of Thoracic Tumor Multimodality Treatment, Cancer Center, West China Hospital, Sichuan University, Chengdu, Sichuan, China.; 7 Department of Radiation Oncology, Cancer Center, West China Hospital, Sichuan University, Chengdu, Sichuan, China.

**Keywords:** radiotherapy, abscopal effect, lenalidomide, dendritic cell cross-presentation

## Abstract

**Background:** Hypofractionated radiotherapy (hRT) can induce a T cell-mediated abscopal effect on non-irradiated tumor lesions, especially in combination with immune checkpoint blockade (ICB). However, clinically, this effect is still rare, and ICB-mediated adverse events are common. Lenalidomide (lena) is an anti-angiogenic and immunomodulatory drug used in the treatment of hematologic malignancies. We here investigated in solid tumor models whether lena can enhance the abscopal effect in double combination with hRT.

**Methods:** In two syngeneic bilateral tumor models (B16-CD133 melanoma and MC38 colon carcinoma), the primary tumor was treated with hRT. Lena was given daily for 3 weeks. Besides tumor size and survival, the dependence of the antitumor effects on CD8^+^ cells, type-I IFN signaling, and T cell costimulation was determined with depleting or blocking antibodies. Tumor-specific CD8^+^ T cells were quantified, and their differentiation and effector status were characterized by multicolor flow cytometry using MHC-I tetramers and various antibodies. In addition, dendritic cell (DC)-mediated tumor antigen cross-presentation *in vitro* and directly *ex vivo* and the composition of tumor-associated vascular endothelial cells were investigated.

**Results:** In both tumor models, the hRT/lena double combination induced a significant abscopal effect. Control of the non-irradiated secondary tumor and survival were considerably better than with the respective monotherapies. The abscopal effect was strongly dependent on CD8^+^ cells and associated with an increase in tumor-specific CD8^+^ T cells in the non-irradiated tumor and its draining lymph nodes. Additionally, we found more tumor-specific T cells with a stem-like (TCF1^+^ TIM3^-^ PD1^+^) and a transitory (TCF1^-^ TIM3^+^ CD101^-^ PD1^+^) exhausted phenotype and more expressing effector molecules such as GzmB, IFNγ, and TNFα. Moreover, in the non-irradiated tumor, hRT/lena treatment also increased DCs cross-presenting a tumor model antigen. Blocking type-I IFN signaling, which is essential for cross-presentation, completely abrogated the abscopal effect. A gene expression analysis of bone marrow-derived DCs revealed that lena augmented the expression of IFN response genes and genes associated with differentiation, maturation (including CD70, CD83, and CD86), migration to lymph nodes, and T cell activation. Flow cytometry confirmed an increase in CD70^+^ CD83^+^ CD86^+^ DCs in both irradiated and abscopal tumors. Moreover, the hRT/lena-induced abscopal effect was diminished when these costimulatory molecules were blocked simultaneously using antibodies. In line with the enhanced infiltration by DCs and tumor-specific CD8^+^ T cells, including more stem-like cells, hRT/lena also increased tumor-associated high endothelial cells (TA-HECs) in the non-irradiated tumor.

**Conclusions:** We demonstrate that lena can augment the hRT-induced abscopal effect in mouse solid tumor models in a CD8 T cell- and IFN-I-dependent manner, correlating with enhanced anti-tumor CD8 T cell immunity, DC cross-presentation, and TA-HEC numbers. Our findings may be helpful for the planning of clinical trials in (oligo)metastatic patients.

## Background

Historically, radiotherapy (RT) was mainly viewed as a directly tumoricidal, local treatment, but more recent preclinical studies have shown that local RT can also elicit a locally and systemically active immune response by acting as *in situ* vaccine 1. Hypofractionated RT (hRT), which delivers the total radiation dose in only a few fractions, appears to be particularly immunogenic 2. The immunogenicity of hRT seems to be largely due to the production of damage-associated molecular patterns such as type I IFN by irradiated tumor cells or dendritic cells (DCs) in the tumor microenvironment 3-5. This facilitates the cross-presentation of tumor antigen by DCs resulting in stimulation of tumor antigen-specific CD8^+^ T cells in the tumor-draining lymph nodes (TDLNs). These newly stimulated T cells proliferate, migrate to the irradiated tumors, and kill tumor cells. In addition, they may cause systemic effects on distant non-irradiated tumor nodules, a phenomenon which is known as "abscopal effect". The abscopal effect of RT has been studied mainly in the context of immune checkpoint blockade (ICB) 6. However, in the clinic, the abscopal effect is not often observed, even in combination with ICB, and many patients experience ICB-induced adverse events 7. Therefore, there is a great interest in identifying alternative combination strategies.

Lenalidomide (lena) is an immunomodulatory imide drug (IMiD), an analogue of the prototypic imide drug thalidomide. IMiDs are used in the clinic to treat hematological malignancies such as multiple myeloma, mantle cell lymphoma, and del(5q) myelodysplastic syndrome 8. Some clinical trials using IMiDs in patients with solid tumors, including in combination with RT in brain tumors 9, have been reported 10-15. Additionally, case reports support the use of this group of drugs in non-hematological tumor types 16, 17. However, so far, there is no FDA approval for IMiDs in solid tumor entities. Whether a combination of RT and lena improves the RT-induced abscopal effect has so far never been investigated.

We have become interested in this group of drugs as they have several potentially interesting anti-tumor effects. IMiDs have anti-angiogenic properties 18, 19. In addition, various immunostimulatory effects of lena have been described, e.g., on DCs treated *in vitro* 20-22 or on T cells 23, including a direct induction of IL-2 in human T cells 24.

High endothelial venules (HEVs) are a type of blood vessel specialized in lymphocyte recruitment and originally found in lymph nodes 25. These cells express the MECA-79 antigen, and can be induced in non-lymphoid tissues at sites of chronic inflammation 26. Tumor-associated HEVs (TA-HEVs) have been found in human tumors, and in mouse tumor models where they positively correlate with CD8^+^ T cell infiltration and better prognosis 27, 28. Moreover, TA-HEVs have recently been associated with a higher proportion of TCF1^+^ TIM3^-^ PD1^+^ stem-like exhausted CD8^+^ tumor-infiltrating lymphocytes (TILs) 28. These TCF1^+^ PD1^+^ cells differentiate into the effector-like “transitory” CD101^-^ TIM3^+^ PD1^+^ subset originally described in the model of lymphocytic choriomeningitis virus (LCMV) 29 and later found in mouse tumor models 30. Terminally differentiated exhausted T cells exhibit expression of CD101 and decreased cytokine production 29-31. Non-terminally differentiated exhausted CD8^+^ T cells were reported to positively correlate with favorable anti-tumor response following immunotherapy 32, 33 or radio/immunotherapy 30, 34, 35.

Here we investigated whether lena can enhance the hRT-induced abscopal effect in two different mouse solid tumor models (the aggressive B16-CD133 melanoma and the MC38 colorectal carcinoma) with established contralateral flank tumors. Although lena monotherapy only slightly delayed tumor growth, lena combined with hRT to the primary tumor improved control of the non-irradiated secondary tumor and survival of mice. This lena-enhanced hRT-mediated abscopal effect was CD8^+^ cell dependent and correlated with enhanced infiltration by tumor-specific CD8^+^ T cells, cross-presenting DCs, and an induction of TA-HEV endothelial cells (TA-HECs) in the non-irradiated secondary tumors.

## Materials and Methods

### Mice and cell lines

All animal experiments were performed in accordance with the German Animal License Regulations and were approved by the animal care committee of the Regierungspräsidium Freiburg (registration number: G21/106). C57BL/6Nrj mice were purchased from Janvier Labs. OT-I mice expressing a transgenic T cell receptor specific for the OVA peptide SIINFEKL presented by H-2K^b^ were provided by Dr. Yakup Tanriver (University of Freiburg, Freiburg, Germany). To generate CD133-expressing melanoma cells (B16-CD133), B16-F10 cells were transduced with lentiviral particles encoding the human stem cell marker CD133 and cultured as described before 36. B16-OVA cells were a gift from Dr. Vincenzo Cerullo (Helsinki). B16-CD133 and B16-OVA tumor cells were cultured in complete RPMI-1640 medium (10% FBS, 100 IU/ml penicillin-streptomycin) at 5% CO_2_, 37 °C. MC38 murine adenocarcinoma cells were purchased from Kerafast, and cultured in complete DMEM medium (10% FBS, 100 IU/ml penicillin-streptomycin) at 8% CO_2_, 37 °C.

### Tumor models and treatment

Cells resuspended in PBS were mixed with Matrigel (Corning) to a 50% final concentration of matrigel; 2x10^5^ B16-CD133 or B16-OVA cells or 5x10^5^ MC38 cells were subcutaneously (s.c.) inoculated into the right flank of the mice. For the abscopal tumors, 2x10^5^ B16-CD133 or B16-OVA cells or 5x10^5^ MC38 cells were additionally injected into the left flank 5 days after the primary tumor injection. When the primary tumor reached 130-200 mm^3^ (B16-CD133 and B16-OVA models) or 180-240 mm^3^ (MC38 model), and secondary tumor volume reached 50 to 100 mm^3^, the mice were randomized to different treatment groups. The primary tumor was irradiated locally with two fractions of 12 Gy (B16-CD133 and B16-OVA models) or three fractions of 8 Gy (MC38 model) on consecutive days. Tumor irradiation was performed using an RS2000 X-ray unit (RadSource). Anesthetized mice were positioned in a custom-made plastic jig with a size-adjustable aperture for the primary tumor; the rest of the mouse body was fully shielded with lead. Dosimetric measurements with phantoms using the thermoluminescent dosimetry method showed that at a distance of 5 mm (from the primary tumor), only 0.06% of the dose delivered is detectable. Lenalidomide (Sigma-Aldrich, 1 mg/mouse) or the vehicle DMSO was injected intraperitoneally (i.p.) into mice every day for 3 weeks. Tumor volume (length × width × height × π/6) was measured three times per week. Survival was defined as the time point after the start of treatment when either the primary or the secondary tumor had reached a size of 1,500 mm^3^. In the CD8 depletion experiment, 200 μg/mouse of anti-CD8 antibodies (clone 2.43, BioXCell) was injected i.p. 3 days before hRT, on the day of the first hRT, and once weekly thereafter. Anti-IFN α/β receptor subunit 1 (IFNAR-1) antibody (250 μg/mouse; clone MAR1-5A3, BioXCell) was given i.p. 3 days before hRT and on the day of the first hRT. Anti-CD70 (200 μg/mouse; clone FR70, BioXCell), anti-CD83 (200 μg/mouse; clone GL-1, Michel-17), and anti-CD86 (200 μg/mouse; clone GL-1, BioXCell) antibodies were injected i.p. every 3 days starting from the day of the first hRT.

### Preparation of single-cell suspensions from tumors and lymphatic organs

Primary and secondary tumors were weighed and digested for 20 min at 37 °C in 5 ml PBS plus MgCl_2_/CaCl_2_ (Gibco; Thermo Fisher Scientific) supplemented with 50 μg/mL DNase I (New England BioLabs) and 120 μg/mL Liberase solution (Roche Life Science). After the incubation, tumor pieces were mechanically ground through a 70-μm strainer (Falcon) and filtered through a 30-μm pre-separation filter (Miltenyi Biotech). Lymph nodes were squeezed through a 70-μm strainer. Red blood cell (RBC) lysis was performed using 1× RBC lysis buffer (eBioscience).

### Flow cytometry analysis

To exclude dead cells, samples were stained with fixable ZombieNIR or ZombieRed (1:1000, BioLegend) or propidium iodide (PI) was added to the samples directly before the measurement. Prior to surface staining, samples were incubated with rat anti-mouse CD16/32 Fc receptor-blocking antibody (clone 2.4G2) for 10 min at 4 °C. M8 tetramer-PE (H-2K^b^, MuLV p15E, KSPWFTTL) and OVA tetramer-PE (H-2K^b^, SIINFEKL) from Baylor College of Medicine (Houston, TX) and CD8-AF700 antibody (KT15, Bio-Rad) were used to detect tumor-specific CD8^+^ T cells. The following anti-mouse antibodies were purchased from BioLegend: CD45-BV510 (clone 30-F11), CD3-PerCP-Cy5.5 (clone 145-2C11), PD1-BV421 (clone 29F.1A12), TIM3-BV605 (RMT3-23), TNFα-BV421 (clone MP6-XT22), IL-2-APC (clone JES6-5H4), CD11b-Pe-Cy7 (clone M1/70), F4/80-FITC and PE (clone BM8), CD49b-FITC and Pe-Cy7 (clone DX5), CD19-FITC (clone 1D3), CD11c-BV650 (clone N418), CD86-BV421 (clone GL1), Ly6C-PerCP-Cy5.5 (clone HK1.4), CD103-APC (clone 2E7), CD31-Pe-Cy7 (MEC13.3), CD106/VCAM-1-PE (clone 105713), CD70-PerCP-Cy5.5 (clone FR70), CD83-PE (clone Michel-19), CD25-BV421 (clone PC61), CD62L-APC-Cy7 (clone MEL-14), CD127-BV421 (clone A7R34); from eBioscience: CD101-Pe-Cy7 (clone Moushi101), GzmB-PE (clone NGZB), IFNγ-FITC (clone XMG1.2), CD3-FITC (clone 145-2C11), SIINFEKL/H-2K^b^-PE (clone eBio25-D1.16), MECA-79-AF488 (clone MECA-79), IRF8-APC (clone V3GYWCH), CD137-PE-Cy7 (clone 17B5), GR1-APC (clone RB6-8C5), FoxP3-PE (clone FJK-16s), CD44-Super Bright™ 645 (clone IM7), KLRG1-PE-Cy7 (clone 2F1); from BD Biosciences: CD54/ICAM-1-APC (clone 3E2), CD102/ICAM2-BV421 (clone 3C4), CD4-PerCP (clone RM4-4). TIM3-APC (clone REA602) was purchased from Miltenyi Biotec. TCF1-AlexaFluor647 and PacificBlue (clone C63D9) was purchased from Cell Signaling. When needed, cells were fixed with the Foxp3/Transcription Factor Staining Buffer Set (eBiosciences, 00-5523-00) or Intracellular Staining Kit (eBiosciences, 88-8824-00) according to the manufacturer's instructions. Data were acquired using a CytoFlex S Flow Cytometer (Beckman Coulter) with CytExpert 2.4.0.28 software followed by data analysis with FlowJo 10.4 software.

### Bone marrow-derived DC (BMDC) generation *in vitro*

All *in vitro* DC assays were performed using complete RPMI-1640 medium containing 10% FBS (Gibco), 100 IU/ml penicillin-streptomycin (Gibco), 10 mM HEPES (Sigma-Aldrich), 1x NEAA (Gibco), 2 mM L-Glutamine (Gibco), and 50 mM β-mercaptoethanol (Sigma-Aldrich). To generate BMDCs, femurs and tibias of healthy C57BL/6 mice were flushed with PBS and the bone marrow filtrated through a 70-μm strainer (Falcon). Erythrocytes were lysed using eBioscience^TM^ RBC lysis buffer (Invitrogen™). Cells were cultured at 3×10^5^ cells/mL in complete medium supplemented with 40 ng/mL rmGM-CSF (ImmunoTools), and DMSO or 10 μM lena (Sigma-Aldrich) at 37°C in 5% CO_2_. Fresh complete medium supplemented with rmGM-CSF, and DMSO or 10 μM lena was added at day 3 and half of the medium was replaced at day 6. Loosely adherent cells were harvested on day 8-9 and used as BMDCs.

### Cross-priming assay

OT-I cells from spleens of OT-I mice were isolated by using a CD8α^+^ T Cell Isolation Kit (Miltenyi Biotec, Cat. No. 130-104-075) and labeled with carboxyfluorescein succinimidyl ester (CFSE; Invitrogen, Cat. No. 65-0850-84). A total of 5x10^4^ DMSO- or lena-treated BMDCs were incubated with 100 μg/mL OVA protein for 24 h at 37 °C in 5% CO_2_. Thereafter, BMDCs were stimulated for 6 h with 1 μg/mL LPS, and co-cultured with CD8^+^ CFSE-labeled OT-I cells at 2:1 ratio for 4 days in the presence of 50 IU/mL rmIL-2. OT-I cell proliferation was measured by flow cytometry.

### RNA-sequence analysis

Total RNA from BMDCs generated in the presence of DMSO or 10 μM lena was extracted using TRIzol™ Reagent (Invitrogen™). Subsequently, libraries were prepared using the TruSeq Stranded mRNA LT Sample Prep Kit (Illumina, San Diego, CA). RNA-seq and analysis were performed by OE Biotech Co, Ltd (Shanghai, China). Counts were normalized with the transcripts per kilobase million method and displayed as heatmap using pheatmap package (1.0.12) in R software (version 4.3.0).

### *Ex vivo* T cell re-stimulation

To evaluate CD8^+^ T cell effector functions, TILs were re-stimulated *in vitro* for 4h at 37 °C, 5% CO_2_ in RPMI medium with 1 μg/mL M8 peptide (KSPWFTTL, GenScript). During the restimulation, 1× Brefeldin A (BioLegend, 420601) was added. Thereafter, intracellular cytokine staining was performed for flow cytometry analysis.

### Immunohistochemistry

Slices from formalin-fixed paraffin-embedded samples were used and stained with Opal 6-Plex Manual Detection Kit (Akoya, NEL861001KT) and the following primary antibodies: rabbit anti-CD31 (Abcam, EPR17259, 1:100), rabbit anti-MECA-79 (Biolegend, clone MECA-79, 1:50). Immunohistochemistry (IHC) images were acquired using a PerkinElmer Vectra Polaris^TM^ multispectral microscope.

### Statistical analysis

Results are presented as mean ± SEM. To compare two groups, an unpaired two-tailed Student's t-test was conducted. The comparison time point for tumor volume measurements was the time point at which at least one mouse of the compared groups had reached the experimental end point. For multiple comparison, one-way ANOVA followed by Tukey's correction was used. Survival data were compared using the log-rank Mantel-Cox test. Statistical significance was set at *P* < 0.05. Prism version 9.0 (GraphPad) was utilized for all statistical analyses.

## Results

### Lena facilitates the radiation-induced abscopal effect

**Figure 1:**
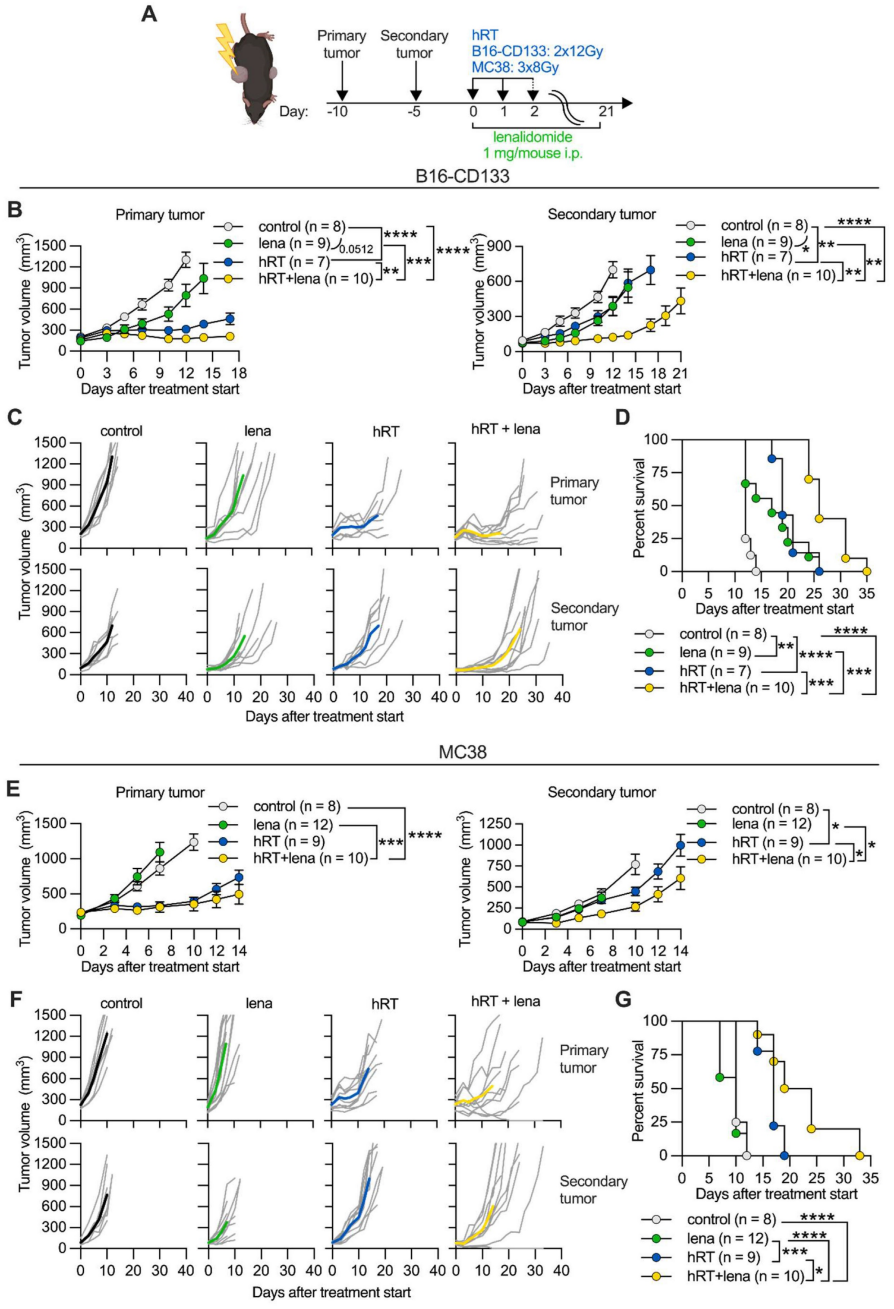
** Adding lena enhances the hRT-induced abscopal effect in the B16-CD133 melanoma and the MC38 colon carcinoma model. A**, Scheme for treatments. **B**,** E**, Growth of primary (left) and secondary (right) B16-CD133 (**B**) and MC38 (**E**) tumors. **C**, **F**, Individual tumor growth curves for primary and secondary B16-CD133 (**C**) and MC38 (**F**) tumors in control mice, or mice treated with lena, hRT, or double therapy. **D**,** G**, Survival of mice bearing contralateral B16-CD133 (**D**) or MC38 (**G**) tumors. Data are presented as mean with SEM and were collected from 3 independent experiments. *P* values (ns, not significant; * *P* < 0.05; ** *P* < 0.01; *** *P* < 0.001) were determined by unpaired two-tailed Student's t-test (B, E), or Kaplan-Meier analysis (D, G).

To investigate whether lena can enhance the hRT-induced abscopal effect, we chose two bilateral flank tumor models in which the primary tumor was irradiated but the secondary was not (**Figure 1A**). Tumor irradiation was done either in two fractions of 12 Gy or three fractions of 8 Gy, based on previous preclinical studies conducted by us 30, 37, 38 and others 2 which showed that these schedules can be immunogenic. Lena or the vehicle DMSO (control) were injected i.p. every day for 3 weeks similar to previously reported studies 19,21.

First, we used the B16-CD133 melanoma model which, by expressing the human tumor stem cell antigen CD133, shows increased immunogenicity compared to the B16 wild-type model 36. B16-CD133 tumor-bearing mice received two fractions of 12 Gy to the primary on two consecutive days. Compared with vehicle control, lena monotherapy only slightly reduced the growth of the secondary tumor (**Figure 1B-C**). hRT alone caused significant growth delay of the irradiated tumor, but, similar to lena monotherapy, only a slight growth delay of the secondary tumor. While hRT/lena combination therapy only slightly improved control of the irradiated tumor compared with hRT alone, it strongly enhanced the abscopal response of the non-irradiated tumor compared with lena alone and with hRT alone (**Figure 1B-C**). Both hRT and lena monotherapy significantly prolonged survival compared with control mice, but dual hRT/lena treatment further considerably enhanced survival compared with the respective monotherapies (**Figure 1D**).

Similar results were observed in the MC38 colon cancer model. MC38 tumor-bearing mice received three fractions of 8 Gy to the primary tumor on three consecutive days. In the MC38 model, lena monotherapy had no effect on the primary and secondary tumor (**Figure 1E-F**), and did not affect survival of the mice (**Figure 1G**). hRT alone caused a significant abscopal effect compared with control mice. Also in this model, hRT/lena dual treatment was considerably better than hRT or lena monotherapy, both in terms of the abscopal response (**Figure 1E-F**) and overall survival (**Figure 1G**).

These results showed that the addition of lena to hRT can significantly improve the hRT-induced abscopal effect and promote survival of the mice in comparison with both lena and hRT monotherapies.

### The hRT/lena-induced abscopal effect depends on CD8^+^ cells

**Figure 2:**
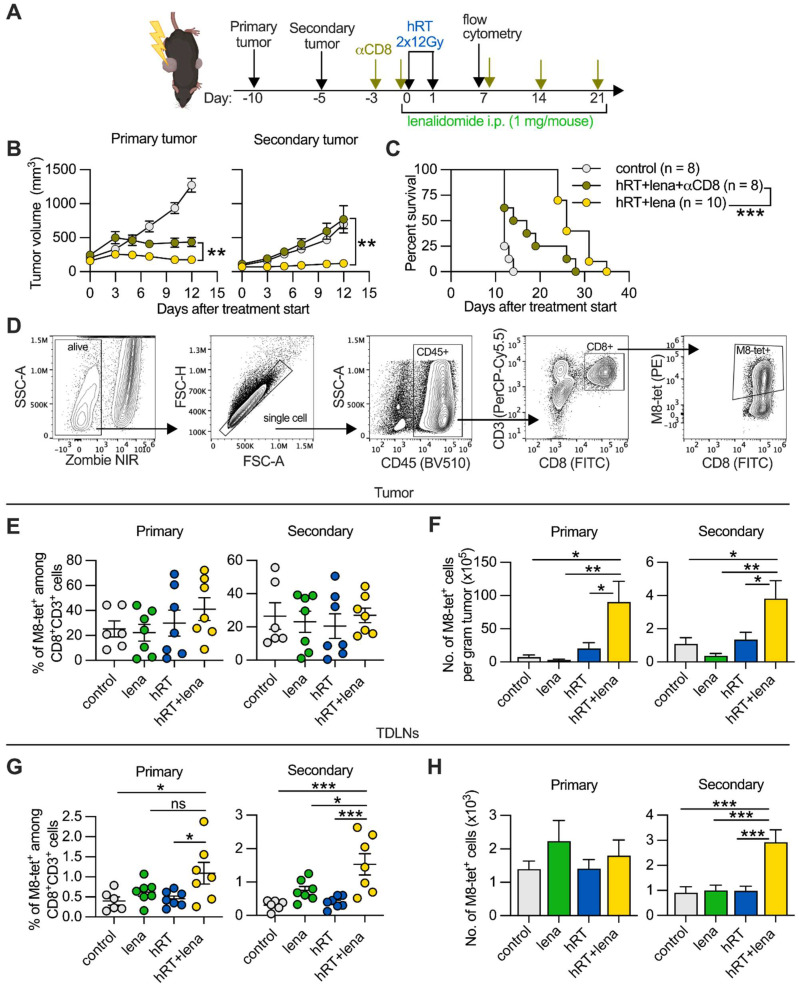
** The enhanced abscopal effect depends on CD8^+^ cells, and adding lena to hRT increases tumor-specific CD8^+^ T cells. A**, Scheme for the depletion of CD8^+^ cells. **B**, B16-CD133 tumor growth of irradiated primary tumor (left) and non-irradiated secondary tumor (right). **C**, Survival of mice. **D**, Gating strategy to identify tumor-specific tetramer^+^ CD8^+^ T cells at day 8 after treatment start. **E-H**, Percentage (E, G), and absolute numbers (F, H) of M8-tetramer^+^ CD8^+^ T cells in the primary and secondary tumors (E, F) and TDLNs (G, H) of control mice (n=6, grey), or mice treated with lena (n=7, green), hRT (n=7, blue) or hRT/lena combination (n=7, yellow). Data are presented as mean with SEM and were collected from 3 independent experiments. *P* values (ns, not significant; * *P* < 0.05; ** *P* < 0.01; *** *P* < 0.001) were determined by unpaired two-tailed Student's t-test (B), Kaplan-Meier analysis (C), or one-way ANOVA with Tukey's multiple comparisons test (E-H).

To investigate mechanisms underlying the superior response to dual therapy, we determined the impact of CD8^+^ T cells. Mice bearing B16-CD133 tumors were treated with hRT/lena and additionally with antibodies to deplete CD8^+^ cells (**Figure 2A**). Control of the irradiated tumor was only partially dependent on CD8^+^ cells (**Figure 2B, left**), presumably because of the strong direct tumoricidal effect of hRT. In contrast, the abscopal response was completely abrogated when CD8^+^ cells were depleted (**Figure 2B, right**). Consistent with this, depletion of CD8^+^ cells considerably reduced survival of hRT/lena-treated mice (**Figure 2C**). Similar results were obtained in the MC38 colon carcinoma model where CD8^+^ cell depletion worsened control of both primary and secondary tumor and survival of the mice (**Figure S1A-B**).

### hRT/lena combination therapy increases the number of tumor-specific CD8^+^ T cells at the abscopal site

The importance of CD8^+^ T cells in hRT/lena-mediated abscopal tumor control was confirmed by analysis of single-cell suspensions of tumors and TDLNs of B16-CD133 tumor-bearing mice at day 8 after treatment start using flow cytometry (**Figure 2D**). In both primary and secondary tumor, hRT/lena treatment caused a considerable increase in the number of bulk CD8^+^ T cells (**Figure S2A-B**). To detect tumor-specific CD8^+^ T cells, we used the M8-tetramer, which recognizes the KSPWFTTL epitope of the MuLV gp70 glycoprotein expressed by various mouse tumor cell lines including B16 melanoma 39 and MC38 colon carcinoma 40. As shown in **Figure 2E-F**, in the B16-CD133 model M8-tetramer^+^ CD8^+^ T cells per gram also increased in both primary and secondary tumor of hRT/lena-treated mice compared to control mice and the respective monotherapies. An increase in tumor-specific CD8^+^ T cells was also found for the MC38 tumor model (**Figure S2C**). In the TDLNs of B16-CD133 tumors, even the percentage of tumor-specific M8-tetramer^+^ CD8^+^ T cells was increased, especially in the lymph nodes draining the secondary tumor (**Figure 2G-H**). Recently, macrophages were shown to mediate the abscopal effect following RT/anti-CD47 treatment 41. However, we did not find differences in anti-tumoral M1 or pro-tumoral M2 macrophages in primary or secondary tumor of hRT/lena-treated compared to hRT-treated mice (**Figure S2D-F**).

These data show that hRT/lena treatment increases the numbers of tumor-specific CD8^+^ T cells, especially in the abscopal tumor, and the increased tumor-specific CD8^+^ T cell frequencies in the TDLNs at both the primary and the secondary sites suggested better CD8^+^ T cell priming or activation.

### hRT/lena therapy enhances DC infiltration of abscopal tumors and lena enhances CD8^+^ T cell priming *in vitro*

**Figure 3:**
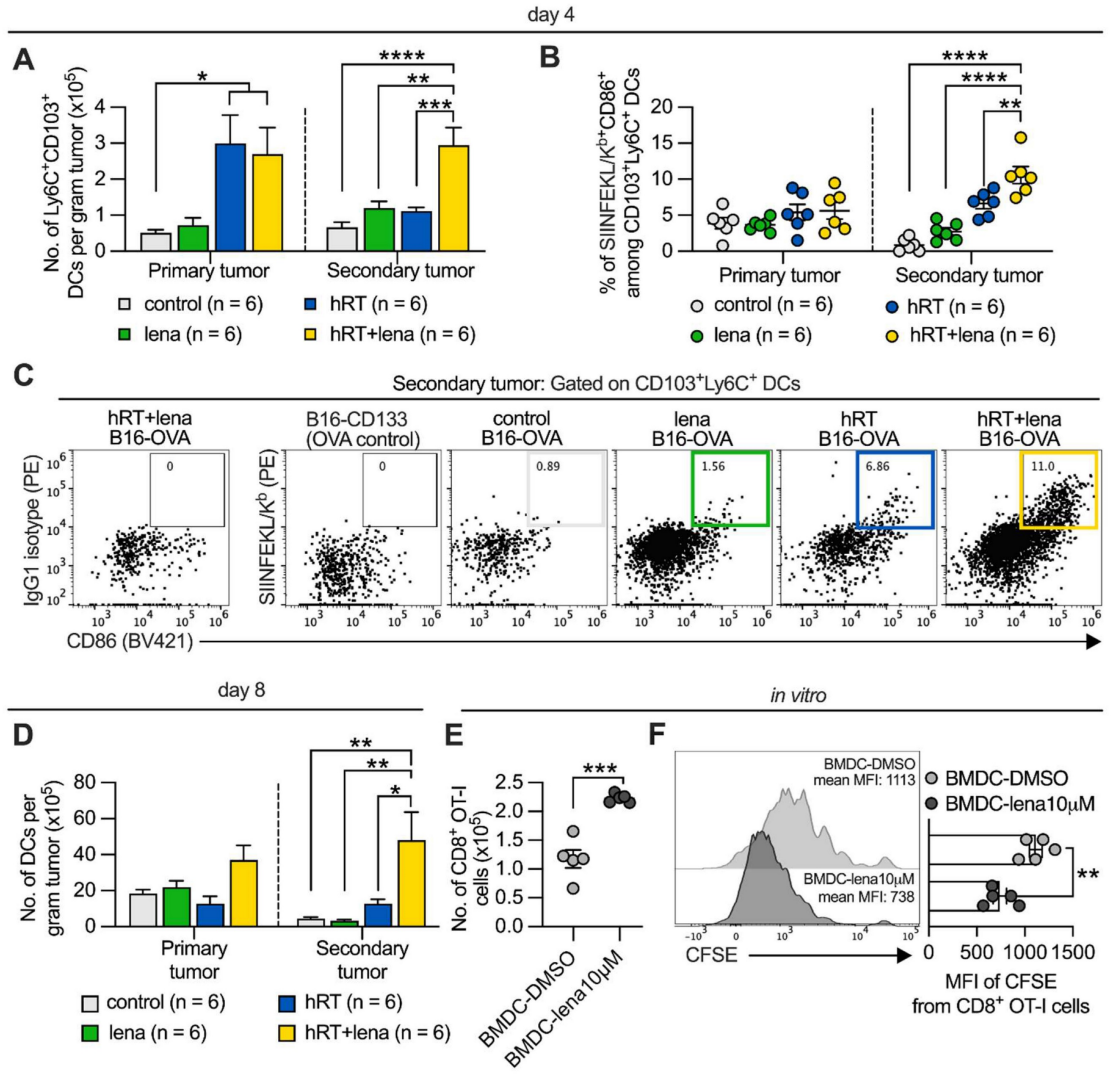
** Lena increases DC numbers in the non-irradiated tumor. A**, Numbers of Ly6C^+^ CD103^+^ DCs per gram tumor in the B16-OVA model at day 4 after treatment start. To identify cross-presenting DCs, first CD3^+^, F4/80^+^, CD49b^+^, and CD19^+^ cells were excluded, then the gate was set on the MHC-II^+^CD11c^+^ population and further on the CD103^+^ Ly6C^+^ subset. **B**, Percentage of mature CD103^+^ Ly6C^+^ CD86^+^ DCs presenting SIINFEKL peptide by H-2K^b^ in the B16-OVA model at day 4 after treatment start. Tumor single-cell suspensions derived from B16-OVA tumors were stained with an IgG1 isotype control antibody as a control for H-2K^b^/SIINFEKL staining, and TILs from B16-CD133 tumors were used as antigen-negative control. **C**, Representative flow cytometry plots for H-2K^b^/SIINFEKL^+^ CD86^+^ cells among CD103^+^ Ly6C^+^ DCs. **D**, Number of total DCs (CD11c^+^ cells) at day 8 after treatment start in the B16-CD133 model. Data are presented as mean with SEM and were collected from 2 independent experiments. **E**, **F**, BMDCs were generated from the BM of healthy mice in the presence of DMSO or 10 µM lena; after 8-9 days, BMDCs were incubated with OVA protein, stimulated with LPS and co-cultured with CFSE-labeled CD8^+^ OT-I T cells. Numbers (E) and proliferation by CFSE dilution (F) of OT-I cells were determined 4 days later. *P* values (ns, not significant; * *P* < 0.05; ** *P* < 0.01; *** *P* < 0.001) were determined by one-way ANOVA with Tukey's multiple comparisons test (A-D) or unpaired two-tailed Student's t-test (E-F).

Cross-presenting DCs typically play a crucial role in priming and activating tumor-specific CD8^+^ T cells by presenting exogenous tumor antigen via MHC class I molecules 42. CD103^+^ Ly6C^+^ DC-like antigen-presenting cells, which are derived from the monocytic lineage, have been suggested to be critical for initiating anti-tumor immune responses early during inflammatory conditions, for example when tumor cells die after chemotherapy or immunotherapy 43, 44. At day 4 after treatment start, we observed an increase in CD103^+^ Ly6C^+^ DCs in the secondary tumor of hRT/lena-treated mice compared to control mice, as well as lena and hRT monotherapy in the B16-OVA model (**Figure 3A**).

To quantify the cross-presentation of a CD8^+^ T cell epitope by DCs, we analyzed B16-OVA single-cell suspensions with a fluorescently labeled antibody specific for SIINFEKL (immunodominant OVA epitope)/H2-K^b^ complexes, a unique agent for epitope-specific and quantitative studies of antigen presentation 45. As shown in **Figure 3B, C**, dual therapy increased the cross-presentation of the SIINFEKL epitope by the CD103^+^ Ly6C^+^ cells in the non-irradiated tumor measured directly *ex vivo*. To confirm these data, we used the B16-CD133 model. In this model, we found a stronger infiltration by total DCs in the abscopal tumor of mice treated with hRT/lena compared to hRT or lena alone at day 8 after therapy start (**Figure 3D**).

We next examined *in vitro* the ability of BMDCs from healthy mice, generated in the presence of 10 μM lena or an equal amount of the diluent DMSO, to cross-prime OVA antigen/SIINFEKL-specific OT-I CD8^+^ T cells. Undifferentiated BMDCs were incubated with OVA protein for 24 h. After exposure to the maturation agent LPS, the DCs were co-cultured for 4 days with naïve CFSE-labeled OT-I cells. BMDCs generated in the presence of lena were more effective at cross-priming naïve OT-I cells, as evidenced by an increase in OT-I cell numbers (**Figure 3E**) and enhanced proliferation reflected by a stronger CFSE signal dilution (**Figure 3F**).

### Tumor control by hRT/lena therapy requires type I IFN signaling and lena enhances expression of IFN response genes and other genes associated with T cell priming in DCs

**Figure 4:**
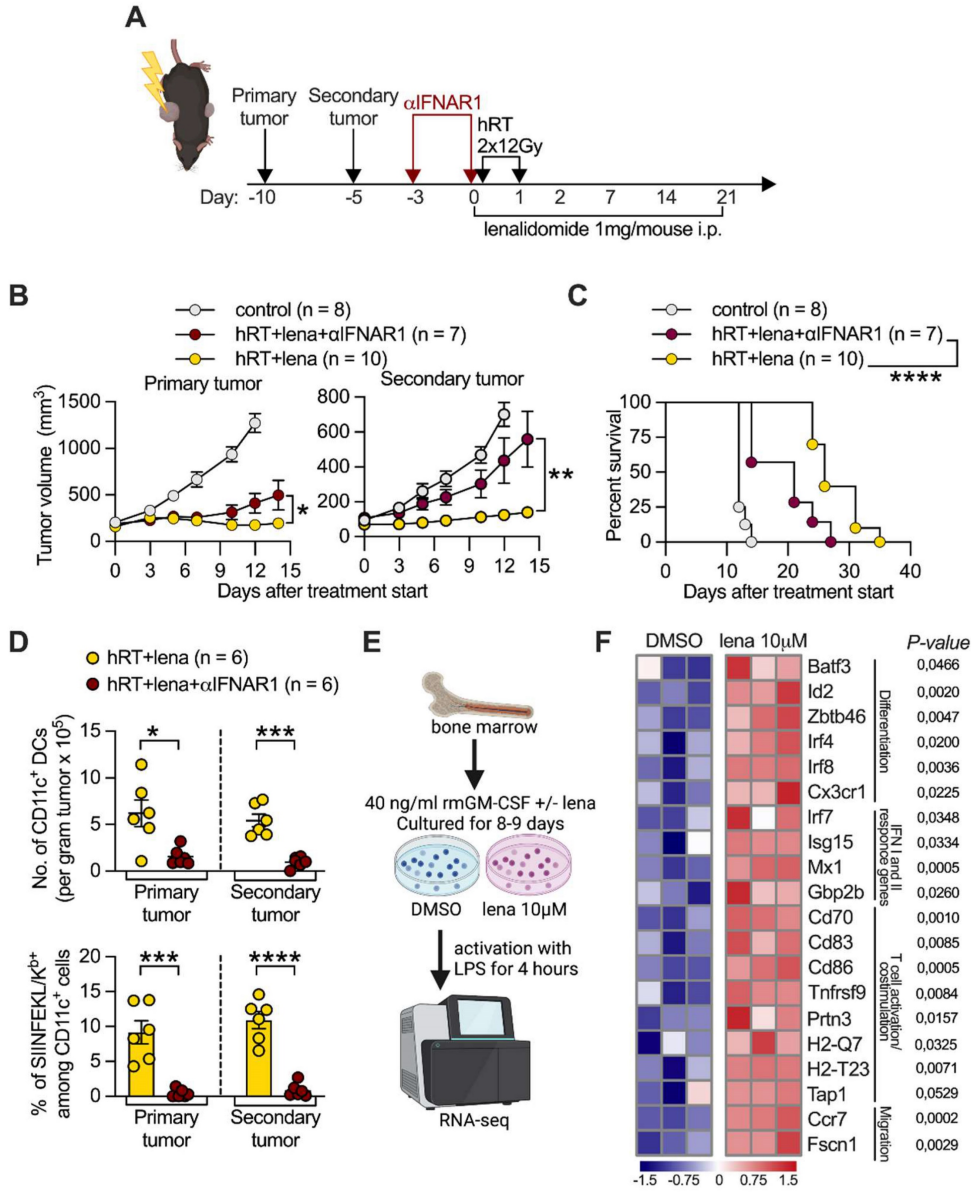
** RT/lena anti-tumor effects depend on type I IFN signaling and lena changes the transcriptional profile of BMDCs. A**, Scheme for treatments. **B**, Growth of irradiated primary (left) and nonirradiated secondary (right) B16-CD133 tumors. **C**, Survival of mice. **D**, Number of CD11c^+^ DCs (upper panel) and % of OVA/SIINKEKL-presenting CD11c^+^ DCs (lower panel) in primary and secondary B16-OVA tumors at day 4 after hRT/lena treatment start with or without type I IFN receptor blockade. **E**, Scheme for the RNA-seq experiment performed on BMDCs which were generated for 8-9 days in the presence of DMSO or 10 μM lena. **F**, Heatmap illustrating the changes in selected genes in BMDCs generated in the presence of either DMSO or lena and stimulated for 4 h with LPS. Data are presented as mean with SEM and were collected from 2 independent experiments (B-D). *P* values (ns, not significant; * *P* < 0.05; ** *P* < 0.01; *** *P* < 0.001; **** P < 0.0001) were determined by unpaired two-tailed Student's t-test (B, D, F), or Kaplan-Meier analysis (C).

Type I IFN signaling is essential for the induction of tumor-specific CD8^+^ T cell responses at the level of DC-mediated CD8^+^ T cell cross-priming/activation 3. To assess whether the efficacy of hRT/lena therapy depends on type I IFN signaling, we administered an IFNα/β receptor-I blocking antibody to mice with B16-CD133 tumors which were undergoing hRT/lena treatment (**Figure 4A**). Blocking the type I IFN receptor only slightly reduced control of the irradiated tumor; however, control of the abscopal tumor and survival of the mice were strongly reduced (**Figure 4B-C**). IFN-I receptor blockade also strongly reduced the density (per gram tumor) of DCs in both primary and secondary tumor in the B16-OVA model (**Figure 4D, upper panel**) and reduced cross-presentation of the immunodominant OVA epitope SIINFEKL by the DCs (**Figure 4D, lower panel**).

**Figure 5:**
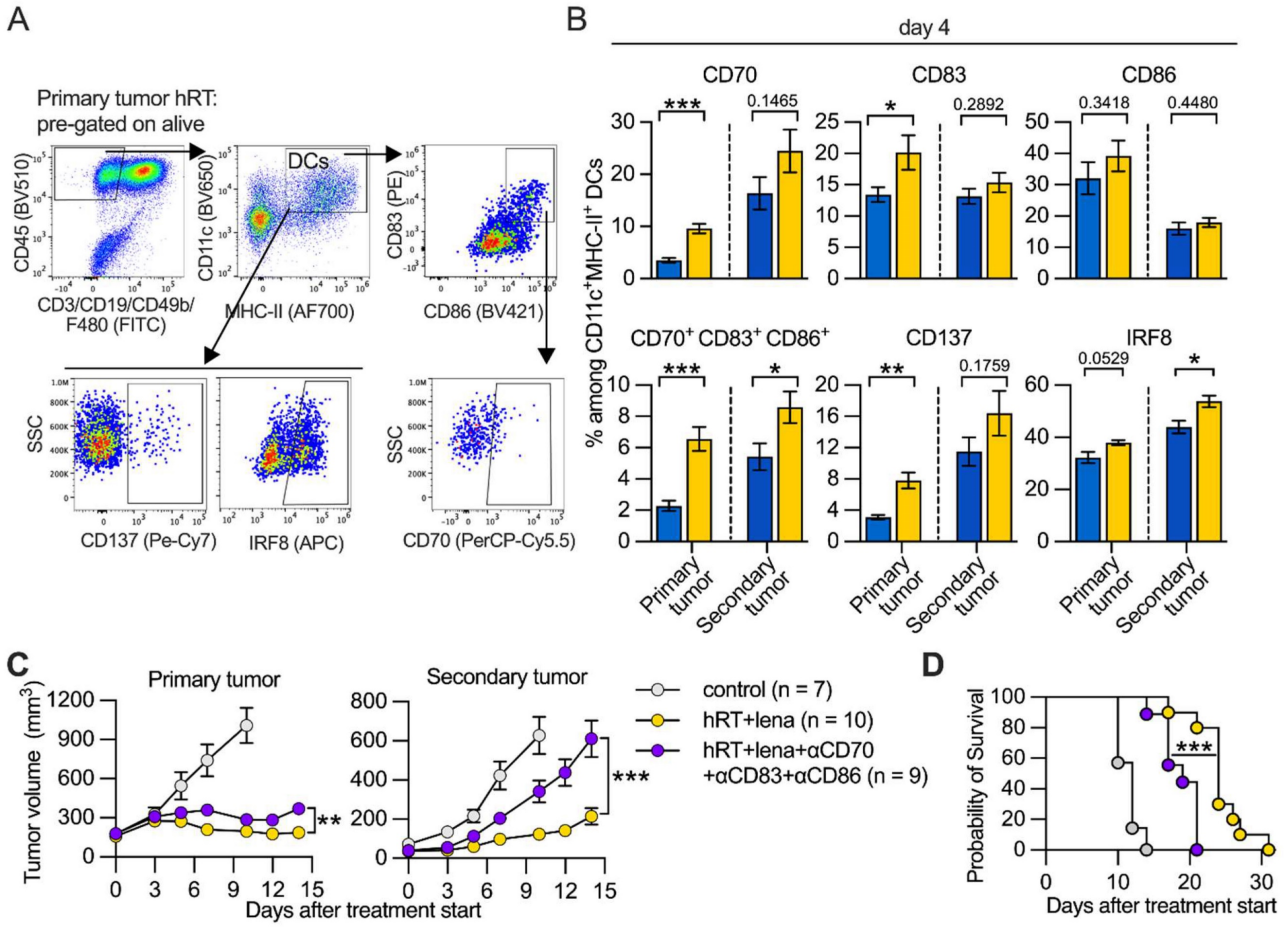
** hRT/lena combination treatment depends on CD70/CD83/CD86 co-stimulatory molecules. A**, Gating strategy. **B**, Percentage of CD70, CD83, CD86, CD137, and IRF8 positive cells among DCs in B16-CD133 melanoma model at day 4 after hRT (blue, n=6) or hRT+lena (yellow, n=6) treatment start. **C**, Growth of irradiated (left) and non-irradiated (right) tumors in untreated mice or hRT/lena-treated mice receiving simultaneous CD70/CD83/CD86 blockade or not (n=7‒10 per group). **D**, Survival of the mice. Data are presented as mean with SEM and were collected from 2 independent experiments. *P* values (ns, not significant; * *P* < 0.05; ** *P* < 0.01; *** *P* < 0.001) were determined by unpaired two-tailed Student's t-test (B, C) or Kaplan‒Meier analysis (D).

RNAseq was used to analyze whether lena affects the expression of genes important for DC-mediated induction of CD8^+^ tumor-specific T cells. For this purpose, DCs were generated from BM of healthy, untreated mice in the presence of lena or the diluent DMSO and matured by a 4-h exposure to LPS (**Figure 4E**). BMDCs generated in the presence of lena showed significantly higher expression of the transcription factor genes *Batf3, Id2, Zbtb46, Irf4, and Irf8*, which like the upregulated *Cx3cr1,* are involved in DC differentiation 46 (**Figure 4F**). They also showed higher expression of genes directly related to DC-mediated activation or priming of CD8^+^ T cells, e.g., genes involved in the type I and II IFN response (*Irf7*, *Isg15*, *Mx1*, and *Gbp2b,*), the maturation of DCs (*Ccr7*, *Cd83*, *Cd86*, *Prtn3*, and *Tnfrsf9*), their migration to lymph nodes (*Ccr7 and Fscn1*), the processing of T cell antigens (e.g., *Tap1*), and the activation and co-stimulation of T cells (*Cd70, Cd83, Cd86*) 47-49. We then wanted to find out whether differentially expressed molecules identified in LPS-matured BMDCs *in vitro* are also upregulated in tumor DCs derived from mice treated with hRT/lena vs. hRT alone. Selected molecules of DCs with importance for CD8^+^ T cell activation/priming were examined in tumor single-cell suspensions by flow cytometry. Individual positive regulatory co-stimulatory molecules (CD70, CD83, CD86) were mainly upregulated in the irradiated tumor of hRT/lena-treated mice. Upregulation in both irradiated and non-irradiated (abscopal) tumor was found when all three co-stimulatory molecules were stained together (CD70^+^ CD83^+^ CD86^+^ DCs) (**Figure 5A-B**). To test if these co-stimulatory molecules played a role in local and abscopal tumor control in hRT/lena-treated mice, we blocked CD70, CD83, and CD86 during hRT/lena treatment by co-administration of blocking antibodies. As shown in **Figure 5C**, this blockade significantly worsened primary and, more prominently, secondary (abscopal) tumor control and diminished overall survival of the mice (**Figure 5D**).

### Addition of lena to hRT increases effector functions of TILs and numbers of stem-like and effector-like exhausted T cells in abscopal tumors

**Figure 6:**
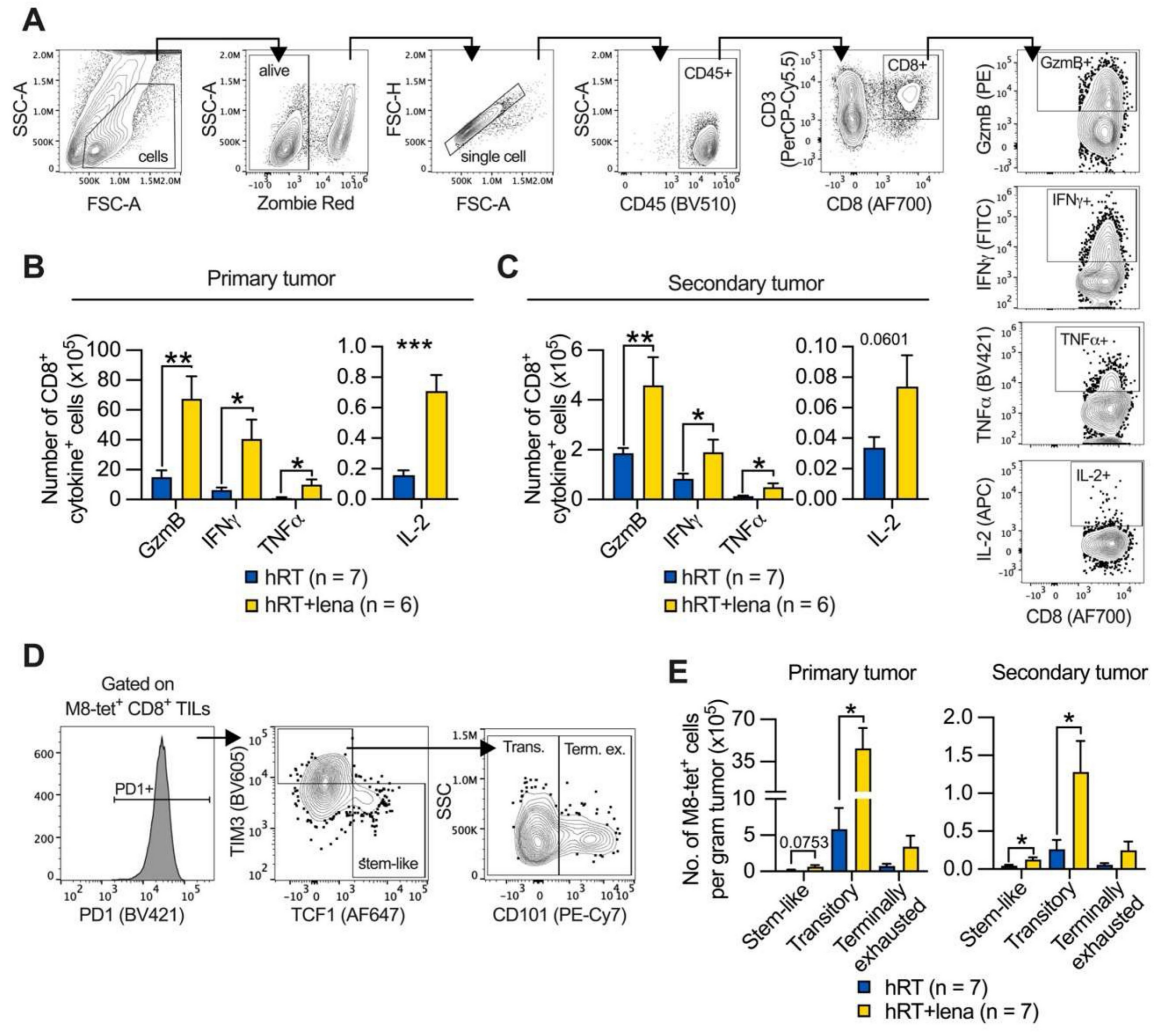
** Adding lena to hRT increases the number of stem- and effector-like exhausted TILs. A**, Gating strategy employed to identify CD8^+^ TILs secreting effector molecules after 4 h *ex vivo* stimulation with M8 peptide and Brefeldin A. **B**,** C**, Numbers of CD8^+^ TILs which secrete GzmB, IFNγ, TNFα, or IL-2 in primary (B), and secondary (C) B16-CD133 tumors at day 8 after treatment start. **D**, Gating strategy to characterize exhausted subsets of tumor-specific T cells. **E**, Cell number of stem-like (TCF1^+^ TIM3^-^ PD1^+^), transitory (CD101^-^ TCF1^-^ TIM3^+^ PD1^+^), and terminally exhausted (CD101^+^ TCF1^-^ TIM3^+^ PD1^+^) M8-tet^+^ CD8^+^ T cells in primary and secondary tumor. Data are presented as mean with SEM and were collected from 3 independent experiments. *P* values (ns, not significant; * *P* < 0.05; ** *P* < 0.01; *** *P* < 0.001) were determined by unpaired two-tailed Student's t-test.

Because we had found a strong increase in bulk and tumor-specific CD8^+^ TILs upon hRT/lena dual treatment compared with all other treatment groups (Figure 2F and Figure S2A), we wanted to determine whether the addition of lena to hRT affects effector functions and exhaustion state of the tumor-specific TILs. Tumor single-cell suspensions (for the composition see **Figure S3A-C**) from hRT- and hRT/lena-treated mice bearing B16-CD133 melanoma tumors were isolated at day 8 after treatment start and re-stimulated *ex vivo* with M8 peptide (the immunodominant epitope of the MuLV p15E tumor antigen presented by H-2K^b^) for 4h in the presence of Brefeldin A. Brefeldin A was added to trap the effector cytokines within the TILs to enable detection by intracellular flow cytometry. The numbers of TILs per gram tumor secreting GzmB, IFNγ, TNFα, and IL-2 were considerably higher in both primary and secondary tumor in the hRT/lena treatment group (**Figure 6A-C**).

In line with the substantially increased density of effector molecule-expressing TILs and better tumor control of irradiated and abscopal tumors (see **Figure 1B**-**C**), analyses of M8-tetramer^+^ CD8^+^ T cell subsets of exhausted cells revealed that the effector-like “transitory” (TIM3^+^ CD101^-^ PD1^+^) population predominantly increased among the tumor-specific TILs of both primary and secondary tumor in hRT/lena-treated compared to hRT-treated mice (**Figure 6D-E**). More undifferentiated “stem-like” (TCF1^+^ TIM3^-^ PD1^+^) TILs also increased significantly in the secondary tumor, but less strongly than transitory cells (**Figure 6E**). In contrast, the terminally differentiated subpopulation of exhausted T cells (TIM3^+^ CD101^+^ PD1^+^) was not significantly increased. Similar results were obtained in the MC38 tumor model (**Figure S4A-B**). In a third model (B16-OVA melanoma), the stem-like subset was significantly increased in the secondary tumor (**Figure S4C**).

Aside from the effects on subsets of the exhaustion lineage, it is important to know the effects of anti-tumor treatments on T cell memory. As shown in **Figure S5A-C**, hRT/lena treatment resulted in a statistically significant increase of tumor-specific CD8^+^ T cells with a memory phenotype (CD44^+^ KLRG1^-^ CD127^+^ CD62L^+^) in the secondary tumor of B16-CD133 tumor-bearing mice compared to hRT monotherapy; in the primary tumor a trend towards increased numbers was observed.

### The addition of lena to hRT increases the frequency of TA-HECs in abscopal tumors

**Figure 7:**
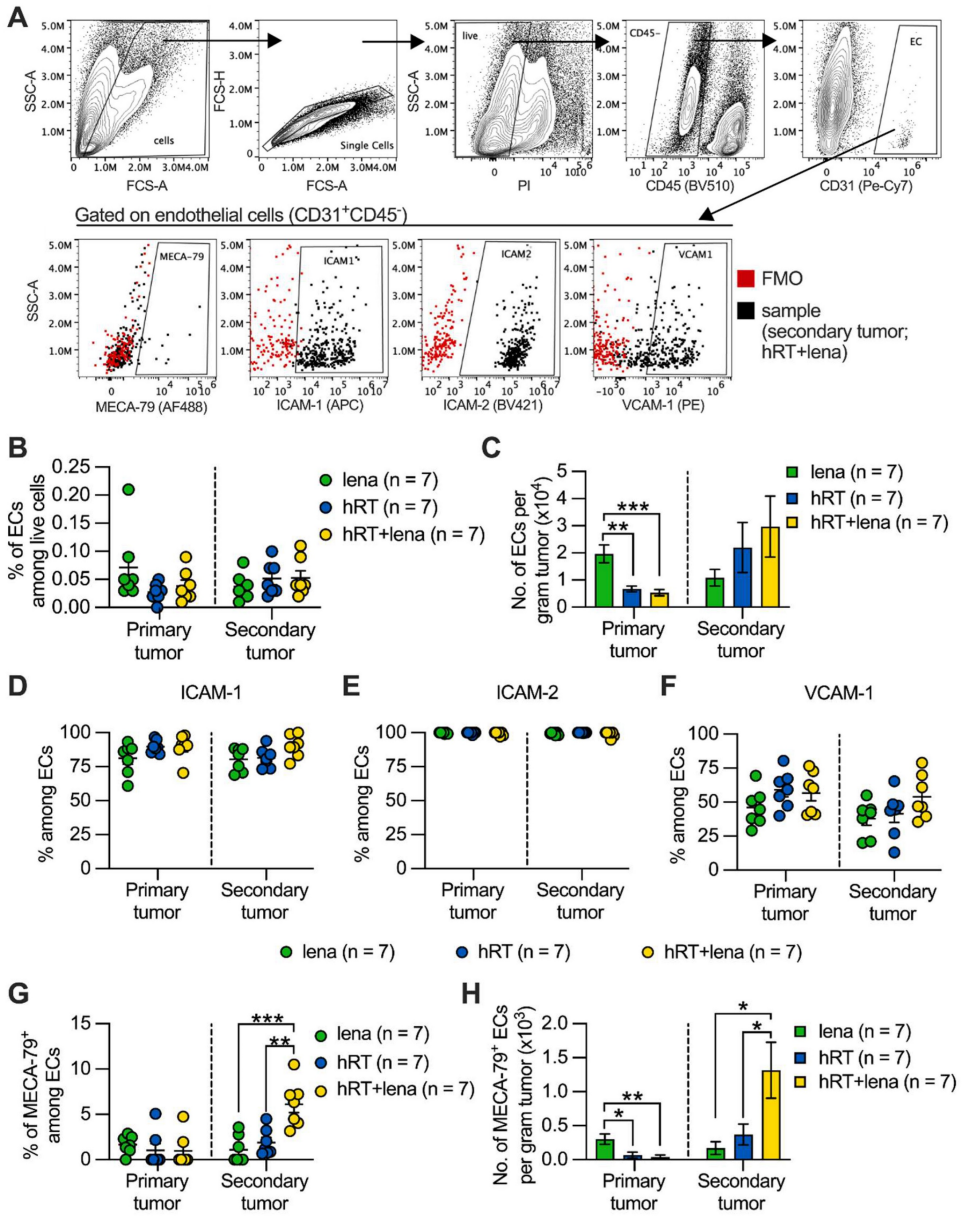
** Combined hRT/lena therapy increases MECA-79^+^ ECs in non-irradiated tumors. A**, Gating strategy. **B**, **C**, Percentage (B) and numbers (C) of ECs (CD31^+^ CD45^-^) in primary and secondary tumor at day 8 after treatment start. **D-F**, Percentage of ICAM-1^+^ (D), ICAM-2^+^ (E), VCAM-1^+^ (F) cells among ECs. **G**, **H**, Percentage (G) and numbers (H) of MECA-79^+^ ECs (TA-HECs). Data are presented as mean with SEM and were collected from 3 independent experiments. *P* values (ns, not significant; * *P* < 0.05; ** *P* < 0.01; *** *P* < 0.001) were determined by one-way ANOVA with Tukey's multiple comparisons test.

IMiDs, including lena, are antiangiogenic and tumor T cell infiltration depends on tumor vascular endothelia. Tumor infiltration by stem-like tumor-specific T cells and ICB response depend on the induction of MECA-79^+^ TA-HEVs 28. These TA-HEVs also express the receptor for CD62L which is not only expressed on naïve but also on memory CD8^+^ T cells (see above) and on subtypes of stem-like exhausted T cells 50. Therefore, we studied the effect of hRT/lena combination therapy on tumor endothelial cells (ECs) by flow cytometry as shown in **Figure 7A**. As shown in **Figure** 7**B**-**C**, no statistically significant difference was found in the percentage and number per gram tumor of CD45^-^ CD31^+^ ECs among the lena, the hRT, and the hRT/lena treatment groups in the non-irradiated secondary tumor. In the irradiated primary tumor, significantly fewer ECs were found, consistent with previous studies demonstrating that higher doses of ionizing radiation can deplete vascular ECs 51 (**Figure 7B-C**).

Finally, we evaluated the expression of adhesion molecules that play a crucial role in facilitating the entry of immune cells into the tumor microenvironment. No difference was observed in expression of ICAM-1, ICAM-2, and VCAM-1 on the ECs among the lena, the hRT, and the hRT/lena treatment groups (**Figure 7D-F**). In contrast, both percentage and numbers per gram tumor of ECs expressing the TA-HEV marker MECA-79 increased in the non-irradiated secondary tumor of hRT/lena-treated mice compared to both lena- and hRT-treated animals. The total number per gram tumor of MECA-79^+^ ECs decreased in the irradiated primary tumor, probably because of RT-induced apoptosis (**Figure 7G-H**). MECA79^+^ tumor blood vessels corresponding to TA-HEVs were visualized by IHC in tumors of mice treated with hRT/lena. Despite the reduction of tumor ECs detected by flow cytometry, these IHC analyses also showed at least larger intact blood vessels in the irradiated primary tumor (**Figure S6**).

## Discussion

We demonstrate here for the first time that the IMiD lena can considerably enhance the RT-induced abscopal effect in mouse solid tumor models. Therefore, lena could be an alternative to ICB for enhancing immunogenic effects of RT in (oligo)metastatic disease states. The enhanced abscopal effect was dependent on CD8^+^ cells and correlated with increased CD8^+^ tumor-specific T cells and cross-presenting DCs particularly in the non-irradiated tumor. In addition, this new double combination therapy enhanced the numbers of stem-like and effector-like, but not terminally differentiated, exhausted tumor-specific CD8^+^ TILs. It also elevated the numbers of MECA-79^+^ tumor vascular ECs which correspond to TA-HEVs that have recently been shown to enhance tumor infiltration by stem-like exhausted T cells 28. Our data also shed more light on the effects of lena on cross-presenting DCs.

Although lena or other IMiDs have not yet been approved for the treatment of solid tumors in patients, other authors have already observed significant antitumor effects of IMiDs alone or in combination, e.g., with an agonistic 4-1BB-specific mAb (as shown for pomalidomide) 52 or DC vaccines (as shown for lena) 21, in solid-tumor models in mice. Only one of the two solid-tumor models studied by us showed a mild response to lena monotherapy. However, in combination with hRT, a pronounced enhancement of the abscopal effect on the non-irradiated tumor was observed in both mouse models. Our depletion experiments show a strong dependence of the hRT/lena-induced antitumor effect on CD8^+^ cells. Indeed, the number of tumor-specific CD8^+^ T cells was increased in tumor tissue. Moreover, in the lymph nodes draining the primary as well as those draining the secondary tumor even the frequencies of tumor-specific CD8^+^ T cells were increased. The latter suggested better DC-mediated CD8^+^ T cell cross-priming caused by adding lena to primary tumor-directed hRT.

DCs are essential for T cell priming 42. Therefore, we also quantified and functionally characterized tumor-infiltrating cross-presenting DCs, and studied the influence of lena on CD8^+^ T cell priming (in co-cultures of naïve OT-I cells and BMDCs fed with the OVA protein), and gene expression changes in cross-presenting DCs *in vitro*. Our data confirm *in vitro* cross-presentation studies by others with BMDCs generated in the presence of lena 20. In addition, we found in hRT/lena-treated mice an increase in the numbers of cells with markers of cross-presenting DCs (CD103^+^ Ly6C^+^) in the non-irradiated (abscopal) tumor directly *ex vivo*. Moreover, in this DC population, the frequency of cells presenting the immunodominant CD8^+^ T cell epitope of the OVA model antigen was strongly increased. Our data confirm findings by others on IMiD-induced upregulation of molecules associated with DC maturation (CD83, CD86) and migration (CCR7) 20-22. In addition, we found in BMDCs higher expression of a number of genes involved in DC differentiation and of genes associated with DC-mediated T cell priming/stimulation: further genes associated with DC maturation, and genes coding for costimulatory molecules, for molecules relevant for DC migration to lymph nodes, for antigen processing, as well as a number of type I and type II IFN response genes. In line with the upregulation of type I IFN response genes in BMDCs by lena and the known dependence of DC-mediated cross-priming on type I IFN, our antibody blocking experiments showed a strong dependence of the enhanced abscopal effect on IFN-I signaling. We confirmed the lena-induced upregulation of co-stimulatory molecules by analyzing tumor DCs by flow cytometry. Furthermore, we proved the functional importance of co-stimulatory molecules by showing that co-treatment of hRT/lena-treated mice with antibodies blocking CD70, CD83, and CD86 worsened primary and, more prominently, secondary (abscopal) tumor control and diminished overall survival of the mice.

Effects of IMiDs (including lena) on the exhaustion state of T cells are not known so far. In our experiments, the hRT/lena double combination increased stem-like and effector-like, but not terminally differentiated, exhausted CD8^+^ tumor-specific TILs. This increase in non-terminally differentiated exhausted T cells was consistent with the presence of more CD8^+^ TILs secreting effector molecules such as GzmB, IFNγ, TNFα, and IL-2 upon *ex vivo* re-stimulation with epitope-specific peptide. We did not study previously described effects of IMiDs on TNFα production by myeloid cells which has been shown to be reduced 53.

Because of the known anti-angiogenic effects of IMiDs (including lena), we also characterized tumor vascular ECs. Numbers of CD31^+^ ECs did not significantly change in the non-irradiated tumor. Expression levels of the adhesion molecules ICAM-1, ICAM-2, and VCAM-1 on ECs did not change either. However, we found an increase in the number and frequency of MECA-79^+^ CD31^+^ ECs in the non-irradiated tumor of hRT/lena-treated mice. This increase, together with the increase in stem- and effector-like exhausted TILs, is interesting in light of recent observations that MECA-79^+^ ECs facilitate tumor immigration of stem-like exhausted CD8^+^ T cells and subsequent intratumoral expansion of effector-like exhausted cells 28, 54. Of note, MECA-79^+^ TA-HEVs have been shown to be induced by a combination of ICB and anti-VEGFR2 antibodies which like IMiDs are antiangiogenic 55. The concerted increase in tumor-infiltrating DCs, non-terminally differentiated exhausted T cells, and MECA-79^+^ TA-HECs found in the non-irradiated tumors is also consistent with published findings showing that DCs are required for forming MECA-79^+^ HEVs 56 and that CD8^+^ T cells are important regulators of TA-HEVs in a feed-forward loop 54. We did not find an increase in total numbers of MECA-79^+^ ECs in the irradiated primary tumor of hRT/lena-treated mice likely due to RT-induced apoptosis of vascular ECs in the irradiated tumor. However, the remaining ECs may be sufficient to enable T cell immigration into the irradiated tumors in our study. Potentially detrimental or suboptimal effects of higher-dosed irradiation such as the depletion of vascular ECs could perhaps be reduced by using spatially fractionated tumor irradiation 57, 58. However, our IHC analyses show that at least larger blood vessels in the irradiated tumor can be intact 8 days after two 12 Gy fractions. This is consistent with recently published data showing that after tumor irradiation with one fraction of 15 Gy, the structure of larger vessels was not affected, despite substantial apoptosis of ECs found predominantly in smaller vessels 59.

An increase in stem-like tumor-specific CD8^+^ T cells in hRT/lena-treated mice together with an enhanced abscopal effect is interesting in light of recent findings suggesting an important role for stem-like tumor-specific CD8^+^ T cells for inducing the abscopal effect. The proportion of stem-like exhausted cells among subpopulations of tumor-specific CD8^+^ T cells seems to be highest in TDLNs compared to other relevant compartments 30, 60. Co-irradiation of the primary tumor and its draining lymph nodes has been shown to reduce stem-like tumor-specific CD8^+^ T cells at the abscopal site correlating with a reduction in the abscopal effect in RT monotherapy-treated mice 60. In agreement with this, we also found that, among the exhausted CD8^+^ T cell subsets, the stem-like one had the highest expression of CXCR3, a chemokine receptor important for T cell immigration into tumors, and CXCR3 blockade abrogated the abscopal effect 30.

We would like to point out that all the effects including those on tumor-specific T cell responses that we observed here appear to be independent of the known IMiD target cereblon which is an E3 ligase component of the ubiquitin system. In the human system, it has been shown that important IMiD-induced immunomodulatory effects, such as induction of the cytokine IL-2 in T cells, depend on ubiquitin-proteasome-mediated degradation of negative regulatory lymphoid transcription factors with the help of cereblon. This was also shown specifically for lena 24. IMiD-reactive cereblon exists in humans but, due to rodent polymorphisms, not in mice 61. Therefore, it must be assumed that the effects observed here are independent of cereblon.

Taken together, our data demonstrate that the IMiD lena can enhance T cell-mediated effects of hRT, especially the systemic (abscopal) effect on unirradiated tumor lesions. Lena could therefore represent an alternative to ICB for combination with RT to enhance RT-mediated T cell responses. The complete mechanisms responsible for the observed lena-induced changes are currently not clear. Our *in vivo* and *in vitro* data suggest an impact on T cell priming/stimulation. However, the extent to which direct effects on DCs, T cells, or enhanced tumor immigration of T cells through TA-HEVs are important for this, or which other IMiD effects (e.g., antiangiogenic effects) may be important, needs to be further investigated.

## Supplementary Material

Supplementary figures.

## Abbreviations

BMDC: bone marrow-derived DCs
DCs: dendritic cells
ECs: endothelial cells
HEVs: high endothelial venules
hRT: hypofractionated radiotherapy
ICB: immune checkpoint blockade
IMiDs: immunomodulatory imide drugs
LCMV: lymphocytic choriomeningitis virus
lena: lenalidomide
IFNAR-1: IFN α/β receptor subunit 1
IHC: immunohistochemistry
TA-HECs: tumor-associated high endothelial cells
TA-HEVs: tumor-associated high endothelial venules
TDLNs: tumor-draining lymph nodes
TILs: tumor-infiltrating lymphocytes

## References

[1] Formenti SC, Demaria S (2012). Radiation therapy to convert the tumor into an in situ vaccine. Int J Radiat Oncol Biol Phys.

[2] Demaria S, Guha C, Schoenfeld J, Morris Z, Monjazeb A, Sikora A (2021). Radiation dose and fraction in immunotherapy: one-size regimen does not fit all settings, so how does one choose?. J Immunother Cancer.

[3] Fuertes MB, Kacha AK, Kline J, Woo SR, Kranz DM, Murphy KM (2011). Host type I IFN signals are required for antitumor CD8+ T cell responses through CD8alpha+ dendritic cells. J Exp Med.

[4] Deng L, Liang H, Xu M, Yang X, Burnette B, Arina A (2014). STING-Dependent Cytosolic DNA Sensing Promotes Radiation-Induced Type I Interferon-Dependent Antitumor Immunity in Immunogenic Tumors. Immunity.

[5] Zitvogel L, Galluzzi L, Kepp O, Smyth MJ, Kroemer G (2015). Type I interferons in anticancer immunity. Nat Rev Immunol.

[6] Ngwa W, Irabor OC, Schoenfeld JD, Hesser J, Demaria S, Formenti SC (2018). Using immunotherapy to boost the abscopal effect. Nat Rev Cancer.

[7] Postow MA, Sidlow R, Hellmann MD (2018). Immune-Related Adverse Events Associated with Immune Checkpoint Blockade. N Engl J Med.

[8] Ioannou N, Jain K, Ramsay AG (2021). Immunomodulatory Drugs for the Treatment of B Cell Malignancies. Int J Mol Sci.

[9] Hipp SJ, Goldman S, Kaushal A, Krauze A, Citrin D, Glod J (2020). A phase I trial of lenalidomide and radiotherapy in children with diffuse intrinsic pontine gliomas or high-grade gliomas. J Neurooncol.

[10] Segler A, Tsimberidou AM (2012). Lenalidomide in solid tumors. Cancer Chemother Pharmacol.

[11] Ganesan P, Piha-Paul S, Naing A, Falchook G, Wheler J, Fu S (2014). Phase I clinical trial of lenalidomide in combination with sorafenib in patients with advanced cancer. Invest New Drugs.

[12] Bertino EM, McMichael EL, Mo X, Trikha P, Davis M, Paul B (2016). A Phase I Trial to Evaluate Antibody-Dependent Cellular Cytotoxicity of Cetuximab and Lenalidomide in Advanced Colorectal and Head and Neck Cancer. Mol Cancer Ther.

[13] Harvey RD, Carthon BC, Lewis C, Hossain MS, Zhang C, Chen Z (2020). Phase 1 safety and pharmacodynamic study of lenalidomide combined with everolimus in patients with advanced solid malignancies with efficacy signal in adenoid cystic carcinoma. Br J Cancer.

[14] Reid EG, Shimabukuro K, Moore P, Ambinder RF, Bui JD, Han S (2022). AMC-070: Lenalidomide Is Safe and Effective in HIV-Associated Kaposi Sarcoma. Clin Cancer Res.

[15] Warren KE, Vezina G, Krailo M, Springer L, Buxton A, Peer CJ (2023). Phase II Randomized Trial of Lenalidomide in Children With Pilocytic Astrocytomas and Optic Pathway Gliomas: A Report From the Children's Oncology Group. J Clin Oncol.

[16] Pallotti MC, Nannini M, Agostinelli C, Leoni S, Scioscio VD, Mandrioli A (2014). Long-term durable response to lenalidomide in a patient with hepatic epithelioid hemangioendothelioma. World J Gastroenterol.

[17] Mattes MJ, Mattes JA, Groisberg R, Mattes MD (2021). Therapy of Angiosarcoma with Thalidomide and Lenalidomide. Case Rep Oncol.

[18] Lu L, Payvandi F, Wu L, Zhang LH, Hariri RJ, Man HW (2009). The anti-cancer drug lenalidomide inhibits angiogenesis and metastasis via multiple inhibitory effects on endothelial cell function in normoxic and hypoxic conditions. Microvasc Res.

[19] Leuci V, Maione F, Rotolo R, Giraudo E, Sassi F, Migliardi G (2016). Lenalidomide normalizes tumor vessels in colorectal cancer improving chemotherapy activity. J Transl Med.

[20] Henry JY, Labarthe MC, Meyer B, Dasgupta P, Dalgleish AG, Galustian C (2013). Enhanced cross-priming of naive CD8+ T cells by dendritic cells treated by the IMiDs(R) immunomodulatory compounds lenalidomide and pomalidomide. Immunology.

[21] Vo MC, Nguyen-Pham TN, Lee HJ, Jaya Lakshmi T, Yang S, Jung SH (2017). Combination therapy with dendritic cells and lenalidomide is an effective approach to enhance antitumor immunity in a mouse colon cancer model. Oncotarget.

[22] Lopez-Relano J, Martin-Adrados B, Real-Arevalo I, Lozano-Bartolome J, Abos B, Sanchez-Ramon S (2018). Monocyte-Derived Dendritic Cells Differentiated in the Presence of Lenalidomide Display a Semi-Mature Phenotype, Enhanced Phagocytic Capacity, and Th1 Polarization Capability. Front Immunol.

[23] Luptakova K, Rosenblatt J, Glotzbecker B, Mills H, Stroopinsky D, Kufe T (2013). Lenalidomide enhances anti-myeloma cellular immunity. Cancer Immunol Immunother.

[24] Kronke J, Udeshi ND, Narla A, Grauman P, Hurst SN, McConkey M (2014). Lenalidomide causes selective degradation of IKZF1 and IKZF3 in multiple myeloma cells. Science.

[25] Girard JP, Moussion C, Forster R (2012). HEVs, lymphatics and homeostatic immune cell trafficking in lymph nodes. Nat Rev Immunol.

[26] Blanchard L, Girard JP (2021). High endothelial venules (HEVs) in immunity, inflammation and cancer. Angiogenesis.

[27] Martinet L, Garrido I, Filleron T, Le Guellec S, Bellard E, Fournie JJ (2011). Human solid tumors contain high endothelial venules: association with T- and B-lymphocyte infiltration and favorable prognosis in breast cancer. Cancer Res.

[28] Asrir A, Tardiveau C, Coudert J, Laffont R, Blanchard L, Bellard E (2022). Tumor-associated high endothelial venules mediate lymphocyte entry into tumors and predict response to PD-1 plus CTLA-4 combination immunotherapy. Cancer Cell.

[29] Hudson WH, Gensheimer J, Hashimoto M, Wieland A, Valanparambil RM, Li P (2019). Proliferating Transitory T Cells with an Effector-like Transcriptional Signature Emerge from PD-1(+) Stem-like CD8(+) T Cells during Chronic Infection. Immunity.

[30] Onyshchenko K, Luo R, Guffart E, Gaedicke S, Grosu AL, Firat E (2023). Expansion of circulating stem-like CD8(+) T cells by adding CD122-directed IL-2 complexes to radiation and anti-PD1 therapies in mice. Nat Commun.

[31] Philip M, Fairchild L, Sun L, Horste EL, Camara S, Shakiba M (2017). Chromatin states define tumour-specific T cell dysfunction and reprogramming. Nature.

[32] Im SJ, Hashimoto M, Gerner MY, Lee J, Kissick HT, Burger MC (2016). Defining CD8+ T cells that provide the proliferative burst after PD-1 therapy. Nature.

[33] Miller BC, Sen DR, Al Abosy R, Bi K, Virkud YV, LaFleur MW (2019). Subsets of exhausted CD8(+) T cells differentially mediate tumor control and respond to checkpoint blockade. Nat Immunol.

[34] Herrera FG, Ronet C, Ochoa de Olza M, Barras D, Crespo I, Andreatta M (2022). Low-Dose Radiotherapy Reverses Tumor Immune Desertification and Resistance to Immunotherapy. Cancer Discov.

[35] Luo R, Onyshchenko K, Wang L, Gaedicke S, Grosu AL, Firat E (2023). Necroptosis-dependent Immunogenicity of Cisplatin: Implications for Enhancing the Radiation-induced Abscopal Effect. Clin Cancer Res.

[36] Hettich M, Lahoti J, Prasad S, Niedermann G (2016). Checkpoint Antibodies but not T Cell-Recruiting Diabodies Effectively Synergize with TIL-Inducing gamma-Irradiation. Cancer Res.

[37] Luo R, Firat E, Gaedicke S, Guffart E, Watanabe T, Niedermann G (2019). Cisplatin Facilitates Radiation-Induced Abscopal Effects in Conjunction with PD-1 Checkpoint Blockade Through CXCR3/CXCL10-Mediated T-cell Recruitment. Clin Cancer Res.

[38] Wang L, Luo R, Onyshchenko K, Rao X, Wang M, Menz B (2023). Adding liposomal doxorubicin enhances the abscopal effect induced by radiation/alphaPD1 therapy depending on tumor cell mitochondrial DNA and cGAS/STING. J Immunother Cancer.

[39] Kershaw MH, Hsu C, Mondesire W, Parker LL, Wang G, Overwijk WW (2001). Immunization against endogenous retroviral tumor-associated antigens. Cancer Res.

[40] Ye X, Waite JC, Dhanik A, Gupta N, Zhong M, Adler C (2020). Endogenous retroviral proteins provide an immunodominant but not requisite antigen in a murine immunotherapy tumor model. Oncoimmunology.

[41] Nishiga Y, Drainas AP, Baron M, Bhattacharya D, Barkal AA, Ahrari Y (2022). Radiotherapy in combination with CD47 blockade elicits a macrophage-mediated abscopal effect. Nat Cancer.

[42] Wculek SK, Cueto FJ, Mujal AM, Melero I, Krummel MF, Sancho D (2020). Dendritic cells in cancer immunology and immunotherapy. Nat Rev Immunol.

[43] Ma Y, Adjemian S, Mattarollo SR, Yamazaki T, Aymeric L, Yang H (2013). Anticancer chemotherapy-induced intratumoral recruitment and differentiation of antigen-presenting cells. Immunity.

[44] Sharma MD, Rodriguez PC, Koehn BH, Baban B, Cui Y, Guo G (2018). Activation of p53 in Immature Myeloid Precursor Cells Controls Differentiation into Ly6c(+)CD103(+) Monocytic Antigen-Presenting Cells in Tumors. Immunity.

[45] Porgador A, Yewdell JW, Deng Y, Bennink JR, Germain RN (1997). Localization, quantitation, and in situ detection of specific peptide-MHC class I complexes using a monoclonal antibody. Immunity.

[46] Nutt SL, Chopin M (2020). Transcriptional Networks Driving Dendritic Cell Differentiation and Function. Immunity.

[47] Csernok E, Ai M, Gross WL, Wicklein D, Petersen A, Lindner B (2006). Wegener autoantigen induces maturation of dendritic cells and licenses them for Th1 priming via the protease-activated receptor-2 pathway. Blood.

[48] Yamakita Y, Matsumura F, Lipscomb MW, Chou PC, Werlen G, Burkhardt JK (2011). Fascin1 promotes cell migration of mature dendritic cells. J Immunol.

[49] Cabeza-Cabrerizo M, Cardoso A, Minutti CM, Pereira da Costa M, Reis e Sousa C (2021). Dendritic Cells Revisited. Annu Rev Immunol.

[50] Tsui C, Kretschmer L, Rapelius S, Gabriel SS, Chisanga D, Knopper K (2022). MYB orchestrates T cell exhaustion and response to checkpoint inhibition. Nature.

[51] Wijerathne H, Langston JC, Yang Q, Sun S, Miyamoto C, Kilpatrick LE (2021). Mechanisms of radiation-induced endothelium damage: Emerging models and technologies. Radiother Oncol.

[52] Fujiwara Y, Sun Y, Torphy RJ, He J, Yanaga K, Edil BH (2018). Pomalidomide Inhibits PD-L1 Induction to Promote Antitumor Immunity. Cancer Res.

[53] Corral LG, Haslett PA, Muller GW, Chen R, Wong LM, Ocampo CJ (1999). Differential cytokine modulation and T cell activation by two distinct classes of thalidomide analogues that are potent inhibitors of TNF-alpha. J Immunol.

[54] Hua Y, Vella G, Rambow F, Allen E, Antoranz Martinez A, Duhamel M (2022). Cancer immunotherapies transition endothelial cells into HEVs that generate TCF1(+) T lymphocyte niches through a feed-forward loop. Cancer Cell.

[55] Allen E, Jabouille A, Rivera LB, Lodewijckx I, Missiaen R, Steri V (2017). Combined antiangiogenic and anti-PD-L1 therapy stimulates tumor immunity through HEV formation. Sci Transl Med.

[56] Moussion C, Girard JP (2011). Dendritic cells control lymphocyte entry to lymph nodes through high endothelial venules. Nature.

[57] Ferini G, Valenti V, Tripoli A, Illari SI, Molino L, Parisi S (2021). Lattice or Oxygen-Guided Radiotherapy: What If They Converge? Possible Future Directions in the Era of Immunotherapy. Cancers (Basel).

[58] Ferini G, Castorina P, Valenti V, Illari SI, Sachpazidis I, Castorina L (2022). A Novel Radiotherapeutic Approach to Treat Bulky Metastases Even From Cutaneous Squamous Cell Carcinoma: Its Rationale and a Look at the Reliability of the Linear-Quadratic Model to Explain Its Radiobiological Effects. Front Oncol.

[59] Kaeppler JR, Chen J, Buono M, Vermeer J, Kannan P, Cheng WC (2022). Endothelial cell death after ionizing radiation does not impair vascular structure in mouse tumor models. EMBO Rep.

[60] Buchwald ZS, Nasti TH, Lee J, Eberhardt CS, Wieland A, Im SJ (2020). Tumor-draining lymph node is important for a robust abscopal effect stimulated by radiotherapy. J Immunother Cancer.

[61] Chamberlain PP, Lopez-Girona A, Miller K, Carmel G, Pagarigan B, Chie-Leon B (2014). Structure of the human Cereblon-DDB1-lenalidomide complex reveals basis for responsiveness to thalidomide analogs. Nat Struct Mol Biol.

