# Supplementary material for: Hypofractionated radiotherapy combined with lenalidomide improves systemic antitumor activity in mouse solid tumor models
Source: Theranostics. 2024 Apr 8;14(6):2573–88. doi: 10.7150/thno.88864 (PMC11024858; doi:10.7150/thno.88864)
Supplement: Supplementary file 1 — Supplementary figures. [file thnov14p2573s1.pdf]

Supplementary information

Hypofractionated radiotherapy combined with lenalidomide improves systemic antitumor activity in mouse solid tumor models

Kateryna Onyshchenko[1,2,3,4,5,†], Ren Luo[1,2,4,5,6,7,†], Xi Rao[1,4,5], Xuanwei Zhang[6,7], Simone Gaedicke[1], Anca-Ligia Grosu[1,4,5], Elke Firat[1] & Gabriele Niedermann[1, 4, 5,*]

1. Department of Radiation Oncology, Faculty of Medicine, University of Freiburg, Freiburg, Germany
2. Faculty of Biology, University of Freiburg, Freiburg, Germany
3. Laboratory of Biosynthesis of Nucleic Acids, Institute of Molecular Biology and Genetics of NASU, Kyiv, Ukraine
4. German Cancer Consortium (DKTK), Partner Site Freiburg, Freiburg, Germany
5. German Cancer Research Center (DKFZ), Heidelberg, Germany
6. Division of Thoracic Tumor Multimodality Treatment, Cancer Center, West China Hospital, Sichuan University, Chengdu, Sichuan, China
7. Department of Radiation Oncology, Cancer Center, West China Hospital, Sichuan University, Chengdu, Sichuan, China

[†]KO and RL contributed equally.

[*]Correspondence: Gabriele Niedermann, Department of Radiation Oncology, University of Freiburg, Robert-Koch-Strasse 3, 79106 Freiburg, Germany
Tel: +49-76127095140; E-mail: gabriele.niedermann@uniklinik-freiburg.de

Running title: Lenalidomide enhances the RT-induced abscopal effect

Keywords: radiotherapy, abscopal effect, lenalidomide, dendritic cell cross-presentation

Supplementary figure 1

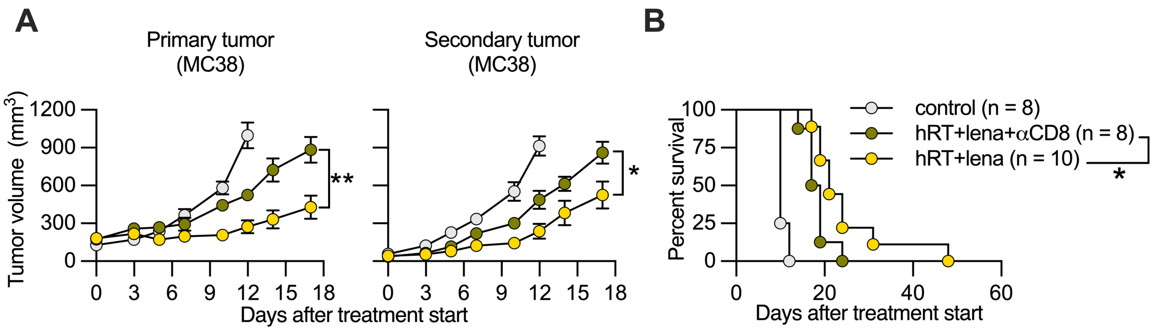

Supplementary figure 1. hRT/lena-mediated local and abscopal tumor control in MC38 colon cancer model depends on CD8$^+$ cells. A. Growth of irradiated primary (left) and nonirradiated secondary (right) MC38 tumors. **B**, Survival of mice. Data are presented as mean with SEM and were collected from 2 independent experiments. *P* values (ns, not significant; * $P < 0.05$; ** $P < 0.01$) were determined by unpaired two-tailed Student's t-test (A) or Kaplan–Meier analysis (B).

Supplementary figure 2

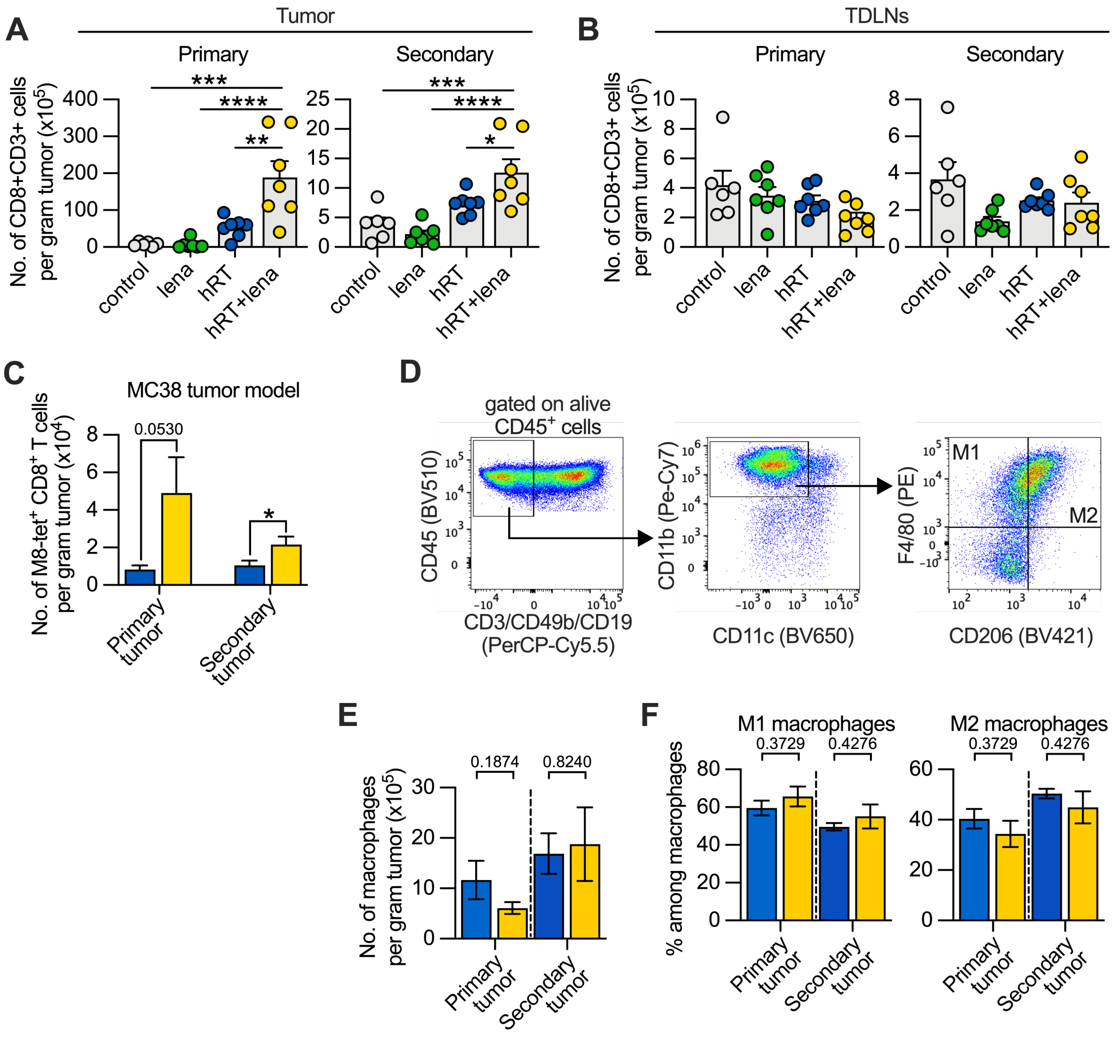

Supplementary figure 2. hRT/lena treatment increases the number of CD8$^+$ T cells in tumors and TDLNs but does not affect macophages. Numbers of bulk CD8$^+$ T cells in primary and secondary tumor (**A**) and TDLNs (**B**) at day 8 after treatment start; untreated control (n=6, grey), lena (n=7, green), hRT (n=7, blue) or hRT/lena (n=7, yellow). **D**, Gating strategy. **E**, Numbers of macrophages (F4/80$^+$ CD11b$^+$) per gram tumor in the B16-CD133 model at day 8 after treatment start. **F**, Percentage of M1 (CD206$^-$) and M2 (CD206$^+$) macrophages in primary and secondary tumors of hRT- (n=7) and hRT+lena (n=7)- treated mice. Data are presented as mean with SEM and were collected from 3 independent experiments. *P* values (ns, not significant; * $P < 0.05$; ** $P < 0.01$; *** $P < 0.001$) were determined one-way ANOVA with Tukey's multiple comparisons test (A, B) or unpaired two-tailed Student's t-test (C, E, F).

Supplementary figure 3

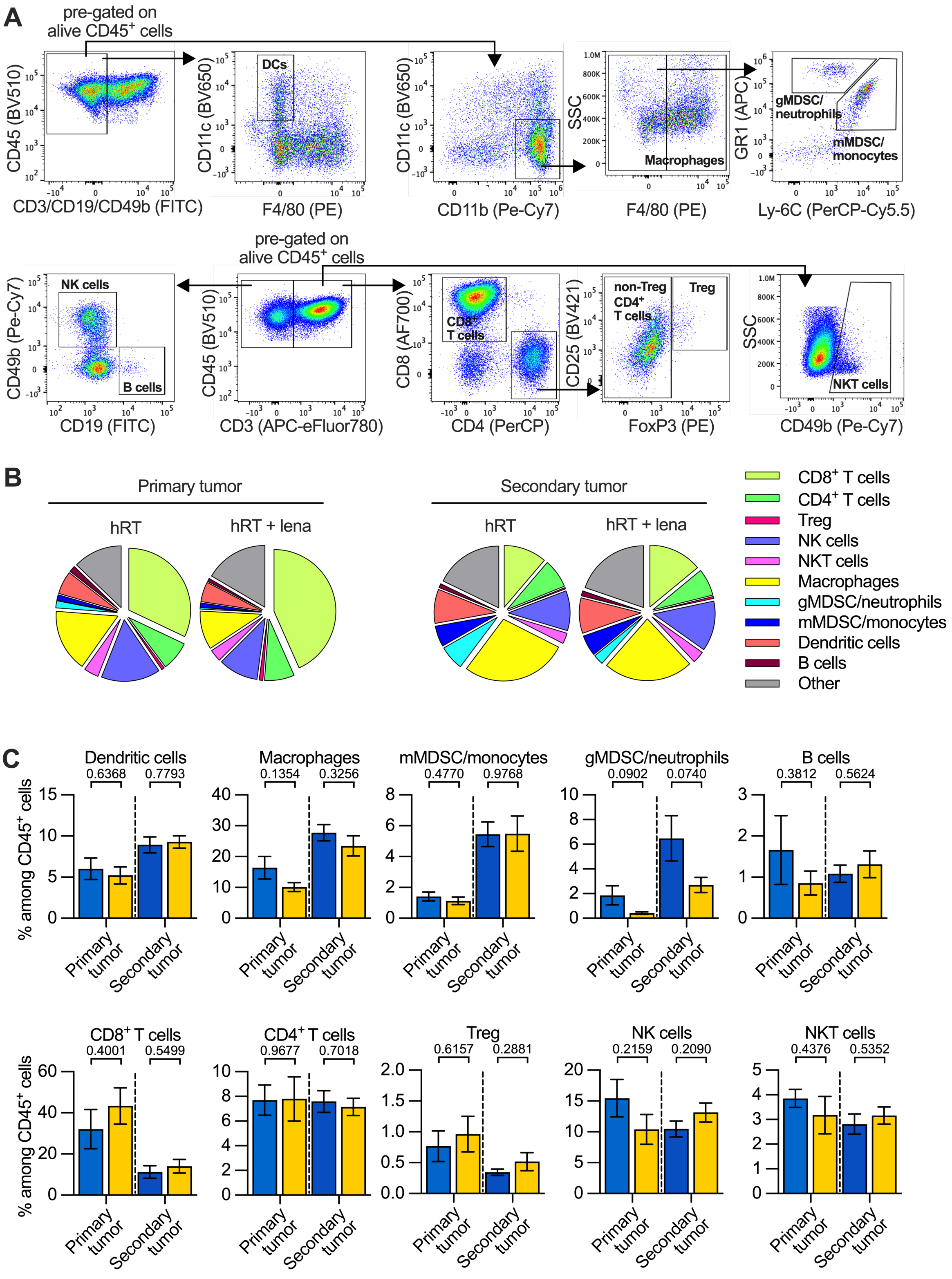

Supplementary figure 3. Lymphoid and myeloid cell composition in primary and secondary tumors of hRT- and hRT+lena-treated B16-CD133 tumor-bearing mice. A, Gating strategy. **B**,

Pie chart summarizing the cell composition within the tumors of hRT- (n=7) and hRT+lena (n=7) -treated mice at day 8 after treatment start. **C**, Percentage of different cell types among CD45$^+$ cells in primary and secondary tumors. Data are presented as mean with SEM and were collected from 3 independent experiments. P values are indicated in the figure and were determined by unpaired two-tailed Student's t-test.

Supplementary figure 4

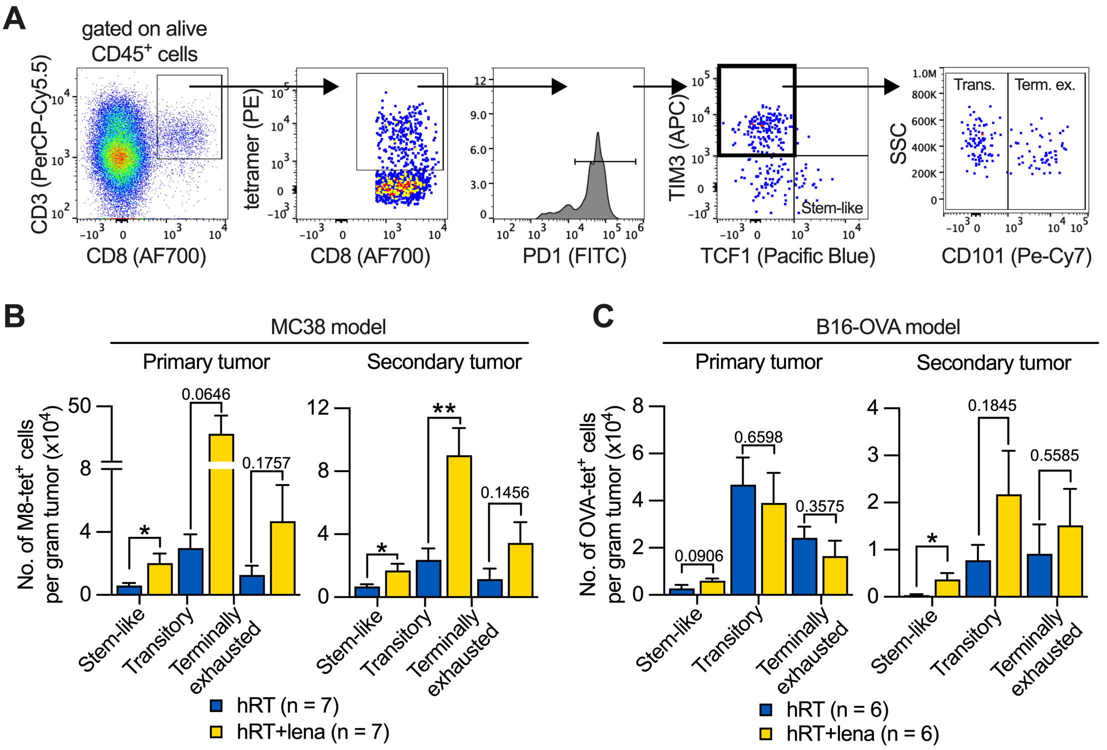

Supplementary figure 4. Adding lena to hRT increases the number of stem- and effector-like exhausted TILs. A, Gating strategy to characterize exhausted subsets of tumor-specific T cells. **B-C**, Cell number of stem-like (TCF1$^+$ TIM3$^-$ PD1$^+$), transitory (CD101$^-$ TCF1$^-$ TIM3$^+$ PD1$^+$), and terminally exhausted (CD101$^+$ TCF1$^-$ TIM3$^+$ PD1$^+$) tetramer$^+$ CD8$^+$ T cells in primary and secondary tumors of MC38 (**B**) and B16-OVA (**C**) tumor-bearing mice. Data are presented as mean with SEM and were collected from 2 independent experiments. P values (ns, not significant; * $P < 0.05$; ** $P < 0.01$) were determined by unpaired two-tailed Student's t-test.

Supplementary figure 5

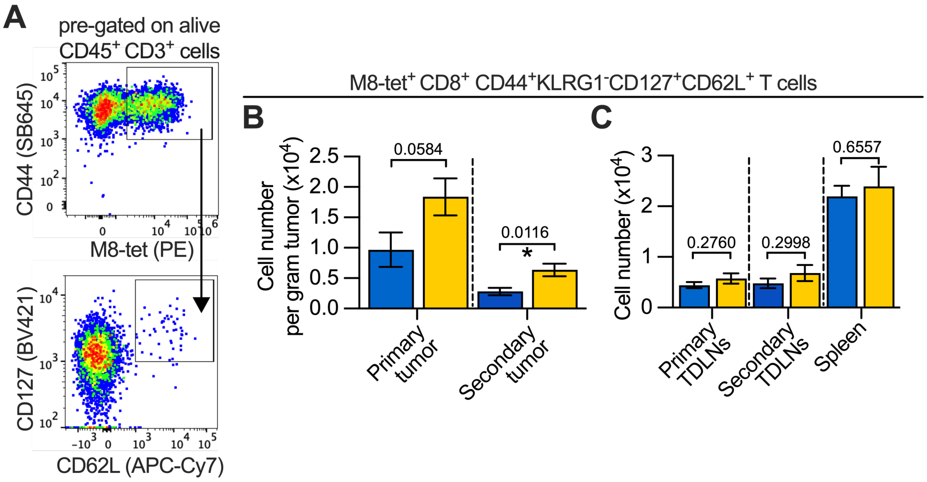

Supplementary figure 5. Adding lena to hRT increases the numbers of CD8^{+} cells with memory phenotype in secondary tumor. **A**, Gating strategy. **B-C**, Numbers of M8-tet^{+} cells with memory phenotype (CD44^{+}KLRG1^{-}CD127^{+}CD62L^{+}) in primary and secondary tumors (**B**), TDLNs, and spleen (**C**) at day 8 after treatment start (n=7 mice per group). Data are presented as mean with SEM and were collected from 3 independent experiments. P values are indicated in the figure and were determined by unpaired two-tailed Student's t-test.

Supplementary figure 6

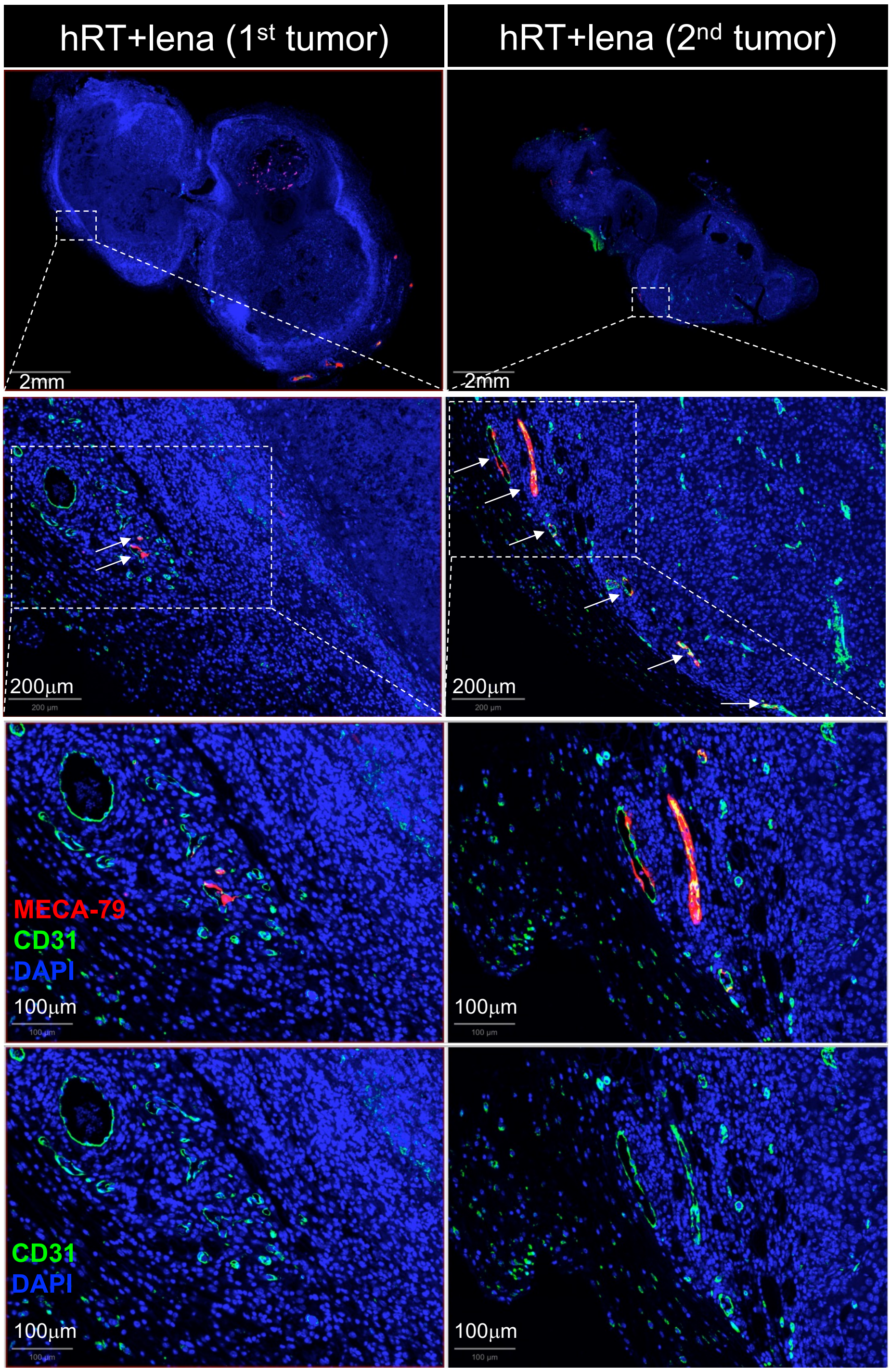

Supplementary figure 6. HEVs in tumors of hRT/lena-treated mice. Representative multiplex immunohistochemistry images showing CD31 (green), MECA-79 (red), DAPI (blue) in primary (left) and secondary (right) tumor of hRT/lena-treated mice at day 8 after treatment start. Arrows indicate MECA-79^{+} vessels (TA-HEVs).
